# Identification of Chemical Components of Qi-Fu-Yin and Its Prototype Components and Metabolites in Rat Plasma and Cerebrospinal Fluid via UPLC-Q-TOF-MS

**DOI:** 10.1155/2021/1995766

**Published:** 2021-12-28

**Authors:** Hengyu Li, Hongwei Zhao, Yong Yang, Dongmei Qi, Xiaorui Cheng, Jiafeng Wang

**Affiliations:** ^1^Innovative Institute of Chinese Medicine and Pharmacy, Shandong University of Traditional Chinese Medicine, Jinan 250355, China; ^2^College of Traditional Chinese Medicine, Shandong University of Traditional Chinese Medicine, Jinan 250355, China

## Abstract

Qi-Fu-Yin, a traditional Chinese medicine formula, has been used to treat Alzheimer's disease (AD, a neurodegenerative disorder) in clinical setting. In this study, the chemical components of Qi-Fu-Yin and its prototype components and metabolites in rat plasma and cerebrospinal fluid, after oral administration, were preliminarily characterized via ultrahigh-performance liquid chromatography coupled with quadrupole time-of-flight tandem mass spectrometry (UPLC-Q-TOF-MS). A total of 180 compounds, including saponins, flavonoids, organic acids, sucrose esters, oligosaccharide esters, phthalides, phenylethanoid glycosides, alkaloids, xanthones, terpene lactones, ionones, and iridoid glycoside, were tentatively characterized. For the first time, 51 prototypical components and 26 metabolites, including saponins, phthalides, flavonoids, sucrose esters, organic acids, alkaloids, ionones, terpene lactones, iridoid glycoside, and their derivatives, have been tentatively identified in the plasma. Furthermore, 10 prototypical components (including butylidenephthalide, butylphthalide, 20(S)-ginsenoside Rh_1_, 20(R)-ginsenoside Rh_1_, and zingibroside *R*_1_) and 6 metabolites were preliminarily characterized in cerebrospinal fluid. These results were beneficial to the discovery of the active components of Qi-Fu-Yin anti-AD.

## 1. Introduction

Traditional Chinese medicine (TCM) plays a vital role in the treatment of various complex chronic diseases owing to the synergistic effects of the formulations and has, accordingly, garnered increasing attention worldwide [1, 2]. Qi-Fu-Yin, a TCM prescription, was first recorded in the book Jingyue Encyclopedia written by Jingyue Zhang during the Ming Dynasty. It is composed of seven herbs—Ginseng Radix et Rhizoma (GRR), Rehmanniae Radix Preparata (RRP), Angelicae Sinensis Radix (ASR), Atractylodis Macrocephala Rhizoma Preparata (ARP), Glycyrrhizae Radix et Rhizoma Preparata cum Melle (GRP), Ziziphi Spinosae Semen (ZSS), and Polygalae Radix Preparata (PRP)—in a ratio of 6 : 9 : 9 : 5 : 3 : 6 : 5 [3]. Qi-Fu-Yin has shown significant effects on Alzheimer's disease (AD) in clinical studies [4, 5]. Owing to its remarkable therapeutic effects and pharmacological activities, Qi-Fu-Yin has attracted the attention of various researchers. Previous studies showed that Qi-Fu-Yin improves the learning ability and memory of rats injected with advanced glycation end products [6, 7] or *β*-amyloid protein [8, 9]. Furthermore, 154 chemical components were unambiguously identified or tentatively characterized in Qi-Fu-Yin using ultrahigh-performance liquid chromatography coupled with quadrupole time-of-flight tandem mass spectrometry (UHPLC-Q-TOF-MS) [10]. However, it remains unknown which components are absorbed into the plasma and brain after oral administration of Qi-Fu-Yin, which hinders the elucidation of its potentially bioactive constituents and the underlying action mechanisms.

**Figure 1:**
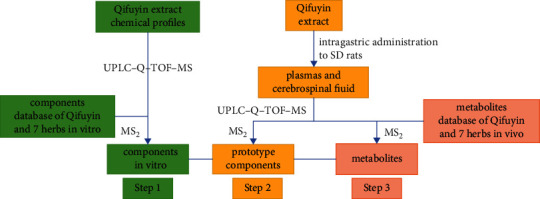
Research strategy for identifying the chemical components in Qi-Fu-Yin, in vitro and in vivo, via UPLC-Q-TOF-MS.

AD is a neurodegenerative disease characterized by the deposition of A*β* and the formation of neurofibrillary tangles in the brain [11]. The ingredients absorbed into blood and that reach a certain concentration can reportedly exert pharmacodynamic effects [12]. The blood-brain barrier (BBB) allows different components to reach the brain and prevents harmful substances from entering the brain. Drugs passing through the BBB can play important roles in brain diseases [13]. Some biotransformed metabolites possess substantial bioactivities and can act as active components [14]. Thus, it is essential to detect components absorbed into blood and elucidate their metabolic profile, which could reveal the pharmacologically active substances and provide potential resources for discovering new drugs from TCM. In this study, a three-step approach based on UHPLC-Q-TOF-MS was implemented to analyze the multicomponent metabolic profiles of Qi-Fu-Yin in rat plasma and cerebrospinal fluid. First, the Qi-Fu-Yin in vitro chemical component database was established by consulting literature on Qi-Fu-Yin and its seven constituent herbs. The components in vitro were identified by their corresponding MS/MS fragment ions in standard solutions and databases. Second, the database of the prototype components was established to characterize the prototypical components in rat plasma and cerebrospinal fluid after oral administration of Qi-Fu-Yin. Under the same LC-MS conditions, the prototype components were identified by comparing the standard solutions, extracts, control, and administered biological samples in parallel. Finally, according to the metabolic pathway and secondary mass spectrometry data of prototype components reported in the literature, the metabolites of Qi-Fu-Yin in plasma and cerebrospinal fluid were tentatively characterized (Figure 1).

## 2. Materials and Methods

### 2.1. Materials and Reagents

GRR, RRP, ASR, ARP, and GRP were purchased from Anxing Traditional Chinese Medicine Co., Ltd. (Anguo, China); ZSS and PRP were purchased from Juyaotang Co., Ltd. (Anguo, China); reference standards of ferulic acid, liquiritin, spinosin, acteoside, 3,6′-disinapoyl sucrose, ginsenoside Rg_1_ (G-Rg_1_), ginsenoside Re (G-Re), ginsenoside Rb_1_ (G-Rb_1_), tenuifolin, and glycyrrhizic acid were purchased from the National Institute for Food and Drug Control (Beijing, China). Acetonitrile and formic acid were of HPLC grade (Fisher, Carlsbad, CA, USA). Deionized water was prepared using a Milli-Q purification system (Millipore, Bedford, MA, USA). Sodium formate was purchased from Waters (Milford, MA, USA).

### 2.2. Preparation of Samples of Qi-Fu-Yin and the Seven Herbs

Qi-Fu-Yin was prepared in the laboratory according to the prescribed protocol [3]. Dried pieces of GRR, RRP, ASR, ARP, GRP, ZSS (crushed), and PRP were accurately weighed and immersed in 9 times amount of water for 30 min; then, the samples were serially decocted with 9 times and 7 times amount of water. After mixing and filtering, the extracts were concentrated to a small volume and lyophilized. An appropriate amount of the lyophilized powder was accurately weighed, dissolved in ultrapure water (equivalent to 50 mg crude drug per mL) in a 25 mL volumetric flask, and mixed evenly via ultrasonication for 30 min. Then, the extracts were centrifuged at 13000 rpm and 4°C for 10 min and filtered through a 0.22 *µ*m membrane. The seven herb samples of Qi-Fu-Yin were prepared in the same manner as the prescribed method.

### 2.3. Animals and Drug Administration

Male SD rats, weighing 200 ± 20 g, were purchased from Beijing Wei Tong Li Hua Experimental Animal Technology Co., Ltd. (Beijing, China). All animal procedures were approved by the Shandong University of Traditional Chinese Medicine Institutional Animal Experimentation Committee (SDUTCM20210119001). All rats were housed at an ambient temperature of 20 ± 1°C with a 12 h light/dark cycle and fed a standard diet and water ad libitum for 3 days before the experiment. The rats were then divided into a control group (orally administered deionized water) and a Qi-Fu-Yin group (orally administered Qi-Fu-Yin) (*n* = 12). To detect the prototype components and metabolites of Qi-Fu-Yin in the rat plasma and cerebrospinal fluid, an 8-fold clinical dosage (1.72 g crude drug per mL, 10 mL per kg, twice daily) was selected as the oral dose [6, 7]. All groups received intragastric administration twice daily for three consecutive days. Before the experiments, the animals fasted for 12 h, with free access to water.

### 2.4. Biological Sample Collection and Preparation

After the last intragastric administration, 500 *μ*L aliquots of serial blood samples were collected from the postorbital venous plexus vein of each rat at 0.5, 1.0, 2, and 4 h. Then, approximately 100 *μ*L of cerebrospinal fluid from each rat was collected at 4 h via percutaneous puncture of the cerebellar medulla cistern [15]. The biological samples collected in heparinized polythene tubes were centrifuged at 3000 rpm at 4°C for 15 min. Subsequently, the supernatant was transferred into new tubes and immediately stored at −80°C before preliminary treatment.

After unfreezing the biological samples in an ice-water mixture, plasma or cerebrospinal fluid was mixed at four different times to enrich the biological samples of each group. To each tube containing 1 mL of plasma or cerebrospinal fluid, 4 mL of methanol was added. The mixture was then vortexed for 2 min and centrifuged at 13000 rpm and 4°C for 10 min. Subsequently, the supernatant was transferred to another tube and dried using sanitary nitrogen gas at room temperature. Then, the residue was redissolved in 100 *μ*L of 30% methanol, vortexed for 2 min, and centrifuged at 13000 rpm and 4°C for 10 min.

### 2.5. UHPLC-Q-TOF-MS Analysis

An ultrahigh-performance liquid chromatography system (ACQUITY H-Class, Waters, Milford, MA, USA) coupled with a Q-TOF (Impact II, Bruker, Bremen, Germany) high-definition mass spectrometer in electrospray ionization mode was used for the chromatographic and mass spectral analyses of all samples. An AMT Halo-C18 column (100 mm × 2.1 mm, 2.7 *μ*m) with a column temperature of 30°C was selected as the separation system. The mobile phase consisted of eluent A (0.1% formic acid in water, v/v) and eluent B (acetonitrile), with a flow rate of 0.30 mL/min. These phases were delivered using a gradient program as follows: 8% B from 0 to 5 min, 8–17% from 5 to 15 min, 17–23% B from 15 to 27 min, 23–35% B from 27 to 43 min, 35–70% B from 43 to 51 min, 70–100% B from 51 to 55 min, and 100% B from 55 to 60 min.

The mass spectra operating parameters were set as follows: capillary voltage of 3.5 kV (ESI+) or −3.0 kV (ESI−), source temperature of 220°C, drying temperature of 220°C, and drying gas flow of 8 L/min. The collision energy was set to range from to 35–75 V for MS/MS acquisition. To ensure mass accuracy and reproducibility, the mass spectrometer was calibrated over a range of 50–1500 Da using a sodium formate solution. All data were processed using Compass Data Analysis^TM^ (V4.4, Bruker, Bremen, Germany).

## 3. Results

### 3.1. In Vitro Chemical Characterization of Qi-Fu-Yin

The base peak chromatograms (BPCs) of Qi-Fu-Yin in the positive and negative ion modes are shown in Figure S1. A total of 180 compounds, including 59 triterpene saponins, 26 flavonoids, 17 organic acids, 16 sucrose esters, 14 oligosaccharide esters, 13 phthalides, 12 phenylethanoid glycosides, 9 alkaloids, 6 xanthones, 3 terpene lactones, 3 ionones, and 2 iridoid glycosides (Table 1), were identified. Twelve compounds were unambiguously identified via comparison with the standard solutions. The structures of other compounds were tentatively characterized based on their retention times, fragmentation pathways, and MS/MS spectra, by referring to the literature.

#### 3.1.1. GRR

Triterpene saponins are the main components of GRR [45]. Ginsenosides can be divided into protopanaxatriol (PPT), protopanaxadiol (PPD), and oleanolic acid (OA) according to their mother skeleton. The diagnostic ions at m/z 475.38, 459.38, and 455.35 corresponded to the PPT, PPD, and OA-type aglycones, respectively. Some special PPT-type ginsenosides were detected at m/z 457.37 owing to dehydration between the 20(21) or 20(22) bonds (Table 1). Continuous or simultaneous loss of different types of glycosyl moieties is another characteristic fragment distribution of ginsenosides. The 132, 146, 162, and 176 Da values indicated the presence of an Ara or Xyl, Rha, Glc, and GlcA glycosyl moiety, respectively. Based on the fragmentation rules, 28 saponins were identified.

Compound 142 produced the adduct ion [M + COOH]^−^ (m/z 1123.5918) and deprotonated molecular ion [M − H]^−^ (m/z 1077.5854), indicating a molecular formula of C_53_H_90_O_22_. Diagnostic ions at m/z 915.5348, 783.4945, 621.4401, and 459.3809 revealed that it was a PPD-type ginsenoside with continuous or simultaneous elimination of Glc and Ara moieties. Thus, compound 142 was assigned to ginsenoside Rc (Table 1). Analogously, PPT-type compounds 79, 84, 88, 104, 118, 123, 131–133, 136, and 137 and PPD-type compounds 139, 142, 144, 145, 149, 170, 174, 176, 178, and 179 were also preliminarily characterized according to their fragmentation pathways and retention times (Table 1). Compounds 158, 164, 165, and 168 had characteristic fragments at m/z 457.37 and were characterized as special PPT-type ginsenosides (Table 1).

Compound 141 only produced a deprotonated molecular ion [M − H]^−^ and diagnostic ions at m/z 455.3527, which indicated that it was an OA-type ginsenoside. Fragmentation ions at m/z 793.4382, 731.4392, 613.3755, and 569.3857 indicated the continuous or simultaneous loss of Glc, GlcA, and CO_2_. Similarly, compounds 148 and 169 were tentatively assigned (Table 1).

#### 3.1.2. RRP

Iridoid glycosides are considered the main components of RRP. The negative ion mode was selected to characterize the RRP components because the fragmentation pathway of glycosyl was easier to detect in the negative ion mode (Figure S1). According to the fragmentation rules, 12 phenylethanoid glycosides, 2 iridoid glycosides, 3 ionone glycosides, and 1 organic acid were identified.

The loss of acyl residues is a characteristic fragmentation pattern of phenylethanoid glycosides. Compound 53 produced a deprotonated molecular ion [M − H]^−^ (m/z 623.1989) in the negative ion mode, which indicated a molecular formula of C_29_H_36_O_15_. The detection of fragmentation ions at m/z 461.1667, 443.1555, and 315.1083 suggested the continuous neutral loss of caffeoyl, H_2_O, and Rha; therefore, compound 53 was identified as acteoside (Figure S2A). Compounds 86 and 92 produced deprotonated molecular ions [M − H]^−^ (m/z 651.23), indicating a molecular formula of C_31_H_40_O_15_. Fragmentation ions at m/z 505.17 and 475.18 corresponded to their neutral loss of Rha and feruloyl. Compounds 86 and 92 were identified as isomartynoside and martynoside, respectively, based on their retention times (Table 1). Other compounds were also preliminarily characterized according to MS_1_/MS_2_ data and retention times available in the literature.

#### 3.1.3. ASR

Organic acids and phthalides are the primary components of ASR, and both can be detected in the positive as well as negative ion modes. The loss of acyl residues in the negative ion mode is characteristic of the fragmentation pattern of organic acids. Phthalides were easily detected by the loss of H_2_O and CO through ring opening in the positive ion mode. According to the fragmentation rules, 14 organic acids and 13 phthalides were identified.

Compound 5 produced a deprotonated molecular ion [M − H]^−^ (m/z 353.0878) in the negative ion mode, indicating a molecular formula of C_16_H_18_O_9_. Fragmentation ions at m/z 191.0563 and 161.0245 indicated the presence of caffeoyl, and the m/z values 155.0350 and 127.0400 indicated the continuous loss of CO and CO_2_. Compounds 13 and 15 were isomers of compound 5. Compounds 5, 13, and 15 were identified as 5-caffeoylquinic acid, chlorogenic acid, and 4-caffeoylquinic acid, respectively, according to the retention time (Table 1).

Alkyl phthalides, such as compound 116 (3-n-butylphthalide), showed abundant protonated molecular ions [M + H]^+^ in the positive ion mode (Table 1). Characteristic fragmentation ions were produced at m/z 173, 155, and 145 because of the continuous or simultaneous neutral loss of H_2_O and CO, while hydroxylated phthalides such as compound 55 (senkyunolide I) showed higher intensities at [M + H−H_2_O]^+^ (Table 1).

#### 3.1.4. ARP

Terpenoids and their lactones are the main components of ARP. Terpene lactones were easily detected by the loss of H_2_O, CO, and C_n_H_2n_ in the positive ion mode. One organic acid and three terpene lactones were identified according to the fragmentation rules.

Compound 175 presented a deprotonated molecular ion [M − H]^−^ (m/z 233.1532) in the positive ion mode, indicating a molecular formula of C_16_H_18_O_9_. Fragmentation ions at m/z 215.1431 and 187.1473 indicated the continuous neutral loss of H_2_O and CO, whereas the m/z values 159.0795, 145.1009, and 131.0848 indicated the continuous neutral loss of C_n_H_2n_; thus, compound 175 was identified as atractylenolide II (Table 1).

#### 3.1.5. GRP

Flavonoids and saponins are the primary components of GRP. Flavonoids have a cyclohexene structure, which readily occurred owing to reverse Diels–Alder (RDA) cleavage in the negative ion mode. Except for the aglycones of compounds 77 and 127, all flavonoids were flavonoid glycosides, which were subdivided into O-glycosides and C-glycosides owing to the different bonding types between glycosyl and aglycones (Table 1). The former can only be detected by the loss of different types of glycosyl groups (Glc, Api, and others), whereas the latter can also be detected by the fragments of C_n_H_2n_O_n_ generated from cross-ring cleavage reactions. Saponins can be easily detected by the characteristic fragments of glucuronic acid residues (GlcA) at m/z 351.05 and 193.03 in the negative ion mode. Seventeen flavonoids, 18 saponins, and 1 organic acid were identified according to the fragmentation rules.

Compound 37 presented an [M − H]^−^ peak at m/z 563.1408, indicating a molecular formula of C_26_H_28_O_14_. Fragmentation ions at the m/z values 503.1197, 473.1098, 443.0992, 413.0882, 383.0778, and 353.0674 indicated the continuous neutral loss of CH_2_O (30 Da); therefore, compound 37 was identified as schaftoside, as shown in Figure S2B. Compound 40 was identified as liquiritin using standard solutions, which presented an [M − H]^−^ peak at m/z 417.1194 and characteristic product ions at m/z 255.0665 with the loss of Glc, and m/z values of 135.0089 and 119.0504 due to RDA cleavage (Table 1). Other flavonoids were identified using data from the literature.

According to the standard solutions, compound 150 was identified as glycyrrhizic acid, which showed [M − H]^−^ at m/z 821.3972, and m/z 803.3855, 777.4059, and 759.3961 due to the simultaneous loss of CO_2_ and H_2_O. Fragmentation ions at m/z 645.3648, 469.3324, 351.0572, and 193.0356 indicated that the mother skeleton was connected to two GlcA groups (Table 1). There were some isomers at m/z 821.39, 823.41, and 837.39 that were preliminarily characterized according to their fragmentation rules and retention times in the literature.

#### 3.1.6. ZSS

Flavonoids and saponins are the main components of ZSS. A total of 10 flavonoids, 2 saponins, 9 alkaloids, and 2 organic acids were identified.

Most of the identified flavonoids contained a structure nucleus of spinosin, and a few of them were the common C-glycosyl flavonoids. Fragmentation ions at m/z 327.08 represented the flavonoid base peak of spinosin in the positive ion mode, and m/z 445.11, 427.10, 325.07, and 307.06 were detected in the negative ion mode (Table 1). Compound 47 was identified as spinosin based on a comparison of standard solutions and presented [M − H]^−^ at m/z 607.1674. Owing to the cross-ring cleavage reaction, characteristic product ions at m/z 487.1252, 367.0823, 337.0722, and 307.0614 were readily observed. In addition, m/z 445.1144 and 427.1039 indicated the neutral loss of Glc and H_2_O, as shown in Figure S2C. Other spinosin flavonoids were identified in the same manner. Common C-glycosyl flavonoids also displayed a neutral loss of C_n_H_2n_O_n_ due to the cross-ring cleavage reaction. Combined with the [M − H]^−^ peak, compounds 28 and 48 were identified as vicenin II and swertisin, respectively (Table 1).

A large number of dammarane-type triterpene glycosides, including inner and outer sugar, were detected in ZSS. The inner sugar was usually Ara (132 Da), whereas the outer sugar generally included Xyl (132 Da), Rha (146 Da), or Glc (162 Da). The characteristic aglycone ions and dehydration products of saponin were easily observed at m/z 455.35 and 437.34, respectively.

Alkaloids can only be detected in the positive ion mode. Compounds 12, 23, and 25 yielded [M]^+^, whereas others produced [M + H]^+^ peaks (Table 1). According to the MS_1_/MS_2_ data, eight isoquinoline alkaloids and one cyclopeptide alkaloid were identified.

#### 3.1.7. PRP

The main components of PRP are xanthones, sucrose esters, oligosaccharide esters, and saponins. Both sucrose esters and xanthones have low molecular weights, whereas oligosaccharide esters and saponins are larger. Based on the fragmentation characteristics of the different types of components, 16 sucrose esters, 14 oligosaccharide esters, 11 saponins, 6 xanthones, and 2 organic acids were identified.

The main characteristic of sugar esters in the negative mode is the neutral loss of acyl (acetyl, feruloyl, p-coumaroyl, sinapoyl, and p-hydroxy benzoyl) residues. For example, compound 90 produced an [M − H]^−^ ion at m/z 767.2416, which corresponds to the molecular formula of C_35_H_44_O_19_. In the MS/MS spectrum, Z_2_^−^ (m/z 529.1567), Z_1_^−^ (m/z 367.1038), ^0,4^X^−^ (m/z 325.0935), ^0,2^X^−^ (m/z 265.0721), Y_2_^−^ (m/z 237.0770), Z_0_^−^ (m/z 205.0507), Y_0_^−^ (m/z 223.0613), and Z_0_^−^−CH_3_ (m/z 190.0271) ions were formed. The presence of Z_2_^−^, Y_2_^−^ and Y_0_^−^, Z_0_^−^ ions indicated the existence of 3,4,5-trimethoxycinnamic acid and sinapoyl, respectively. The presence of Z_2_^−^, Z_1_^−^ and Z_0_^−^ ions indicated that 3,4,5-trimethoxycinnamic acid and sinapoyl moieties were situated on the glucose and fructose residues, respectively. Therefore, compound 90 was deduced to be tenuifoliside C, as shown in Figure S3. The fragmentation rule of oligosaccharide esters was similar to that of sucrose esters. Compound 119 produced an [M − H]^−^ ion at m/z 1349.4019, corresponding to the molecular formula of C_61_H_74_O_34_, whereas the m/z values 1307.3907 and 163.0409, 145.0304 indicated the presence of acetyl and p-coumaroyl, respectively; thus, it was identified as tenuifoliose H (Table 1). The remaining 15 sucrose esters and 13 oligosaccharide esters were characterized on the basis of fragmentation rules and the literature.

The basic structure of saponins in PRP mainly comprised an aglycone substituted at C-3 with a mono-glucosyl saccharide (A-chain) and at C-28 with a second complex oligosaccharide (B-chain). Saponins produced characteristic fragments at m/z 455 and 425 in the negative ion mode because of the easy elimination of CH_2_OH (30 Da) on C-14. For example, compound 107 produced a deprotonated molecular ion [M − H]^−^ (m/z 1103.5328) in the negative ion mode, indicating a molecular formula of C_53_H_84_O_24_. Characteristic fragments were easily observed at m/z 455.3185 [M − H−Glc−H_2_O−CO_2_−Fuc−Rha−Xyl]^−^ and m/z 425.3075 [M−H−Glc−H_2_O−CO_2_−Fuc−Rha−Xyl−CH_2_O]^−^ in the MS/MS spectrum. Therefore, compound 107 was deduced to be polygalasaponin XXVIII (Table 1). According to the fragmentation rules, the remaining 10 saponins were preliminarily characterized.

**Figure 2:**
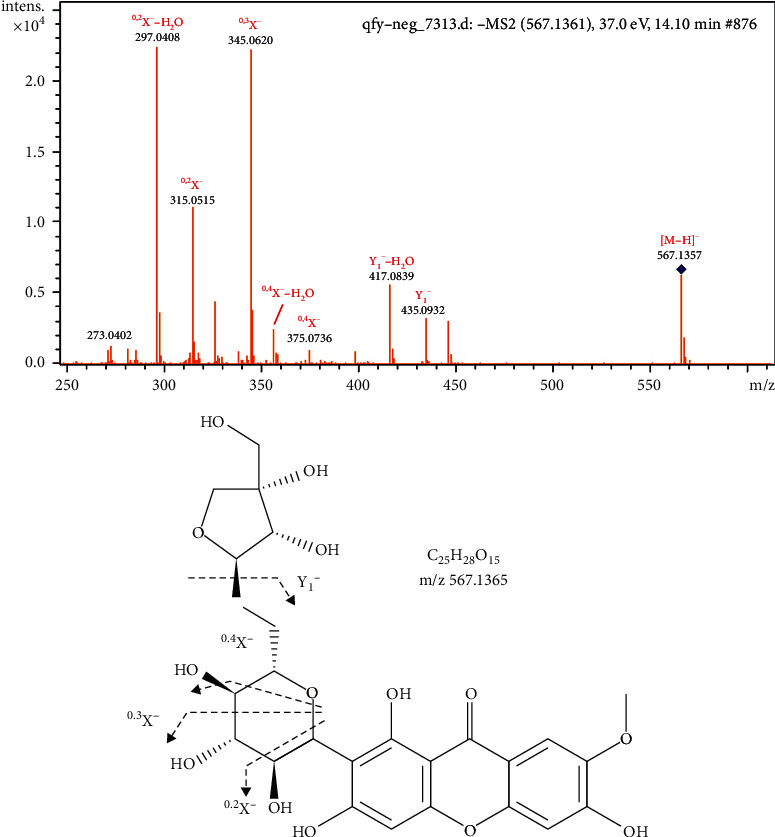
MS/MS spectra and the proposed fragmentation pathways of polygalaxanthone III.

Characteristic fragments of C_n_H_2n_O_n_ were found for xanthones due to cross-ring cleavage. Compound 41 showed a deprotonated molecular [M − H]^−^ ion at m/z 567.1361, indicating a molecular formula of C_25_H_28_O_15_. In the MS/MS spectrum, fragment ions at m/z 435.0932, 417.0839, 375.0736, 357.0621, 345.0620, 327.0518, 315.0515, and 297.0408 corresponded to Y_1_^−^, Y_1_^−^−H_2_O, ^0,4^X^−^, ^0,4^X^−^−H_2_O, ^0,3^X^−^, ^0,3^X^−^−H_2_O, ^0,2^X^−^, and ^0,2^X^−^−H_2_O, respectively. The Y_1_^−^ ions were generated by the loss of Api. The ^0,2^X^−^, ^0,3^X^−^, and ^0,4^X^−^ ions were observed in the MS/MS spectrum, mainly via the cross-ring cleavage reactions in the Glc residue. Therefore, compound 10 was identified as polygalaxanthone III, as shown in Figure 2.

### 3.2. Characterizing the Prototype Components in Plasma after Oral Administration of Qi-Fu-Yin

**Figure 3:**
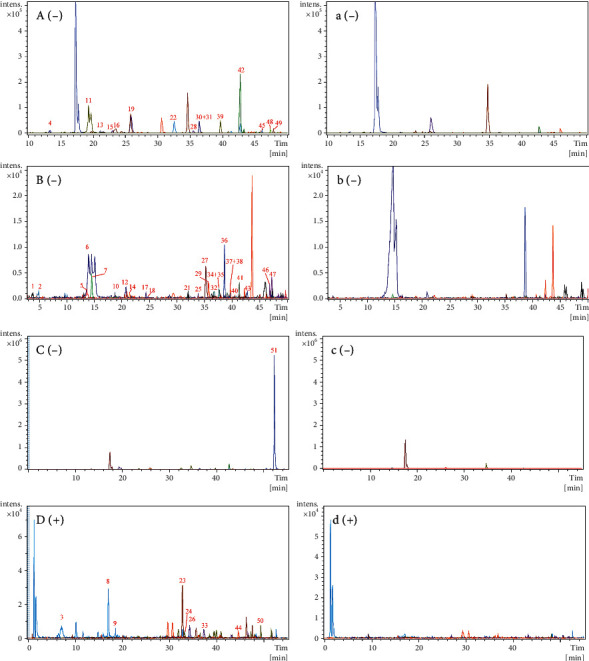
Extracted ion chromatograms (EICs) of prototypical components of Qi-Fu-Yin in the dosed and control plasma in the negative and positive ion modes. (A)–(C) Dosed plasma in the negative mode. (a)–(c) Control plasma in the negative mode. (D) Dosed plasma in the positive mode. (d) Control plasma in the positive mode. Because of the presence of many prototype components in rat plasma, they could not be displayed in the same figure and were, therefore, divided into three panels: (A), (B), and (C).

The identification process for the prototype components was similar to that used in vitro. Using the same UPLC-Q-TOF-MS conditions, 51 prototype components were preliminarily identified by comparing the components of Qi-Fu-Yin in vitro, including 24 triterpene saponins, 10 phthalides, 8 flavonoids, 4 sucrose esters, 1 organic acid, 1 alkaloid, 1 xanthone, 1 terpene lactone, and 1 ionone. Among them, 10 components were compared with the reference standards, and others were identified by comparing the retention times, fragmentation pathways, and MS/MS spectra (Table 2, Figure 3).

**Figure 4:**
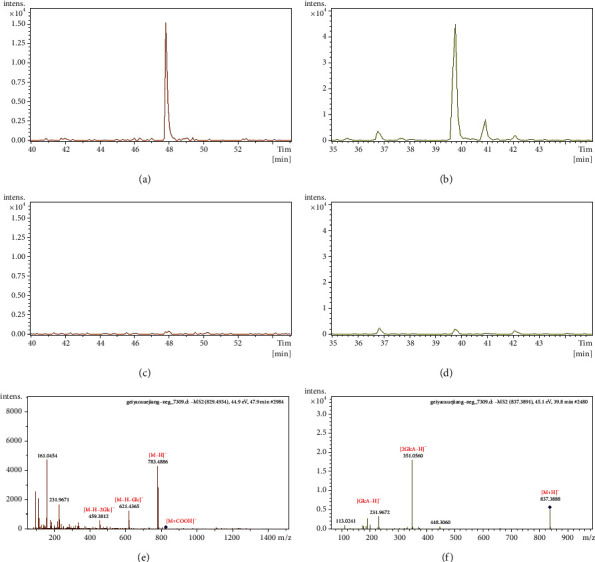
EICs and MS/MS spectra of ginsenoside Rg_3_ and licorice saponin G_2_ in the dosed and control plasma in the negative ion mode. (a) EIC of ginsenoside Rg_3_ in the dosed plasma. (b) EIC of licorice saponin G_2_ in the dosed plasma. (c) EIC of ginsenoside Rg_3_ in the control plasma. (d) EIC of licorice saponin G_2_ in the control plasma. (e) MS/MS spectra of ginsenoside Rg_3_ in the dosed plasma. (f) MS/MS spectra of licorice saponin G_2_ in the dosed plasma.

Some saponins with low molecular weights can be directly absorbed into blood. For example, P53 produced the adduct ion [M + COOH]^−^ (m/z 829.4934) and deprotonated molecular ion [M − H]^−^ (m/z 783.4886), indicating a molecular formula of C_42_H_72_O_13_. Diagnostic ions at m/z 621.4365, 459.3812, and 161.0454 suggested that it was a PPD-type ginsenoside with continuous or simultaneous elimination of Glc moieties. Thus, P53 was assigned to ginsenoside Rg_3_ (Figure 4(a)). P41 produced an [M − H]^−^ peak at m/z 837.3891, indicating a molecular formula of C_42_H_62_O_17_. Furthermore, P41 was identified as glycyrrhizin G_2_ because of the characteristic fragments of glucuronic acid residues, which were readily detected at m/z 351.056 and 193.0351 in the negative ion mode (Figure 4(b)).

Hydroxylated phthalides showed a higher intensity at [M + H−H_2_O]^+^ and were detected by the loss of H_2_O, CO, and C_n_H_2n_ through ring opening in the positive ion mode. For example, P10 and P11 produced [M + H–H_2_O]^+^ at m/z 207.10, and the characteristic fragmentation ions at m/z 189.09, 161.10, and 147.08 indicated neutral loss of H_2_O, CO, and C_3_H_6_. P10 and P11 were identified as senkyunolides I and H, respectively, according to the retention time (Figure S4).

### 3.3. Characterization of Metabolites in Plasma after Oral Administration of Qi-Fu-Yin

**Figure 5:**
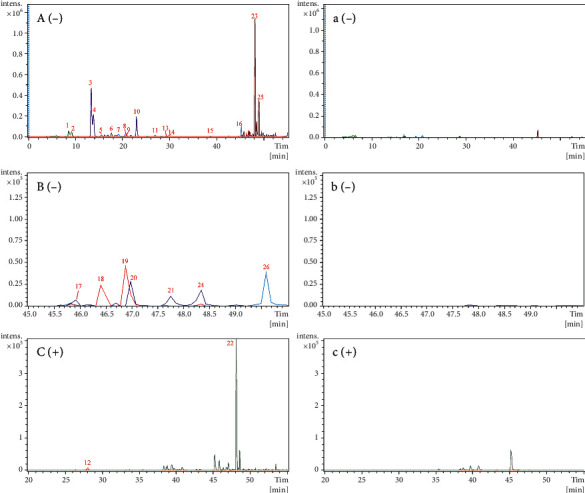
EICs of metabolites of Qi-Fu-Yin in the dosed and control plasma in the negative and positive ion modes. (A)-(B) Dosed plasma in the negative mode. (a)-(b) Control plasma in the negative mode. (C) Dosed plasma in the positive mode. (c) Control plasma in the positive mode. Because of the presence of many metabolites in the rat plasma, they cannot be displayed in the same figure and are, therefore, divided into two panels: (A) and (B).

**Figure 6:**
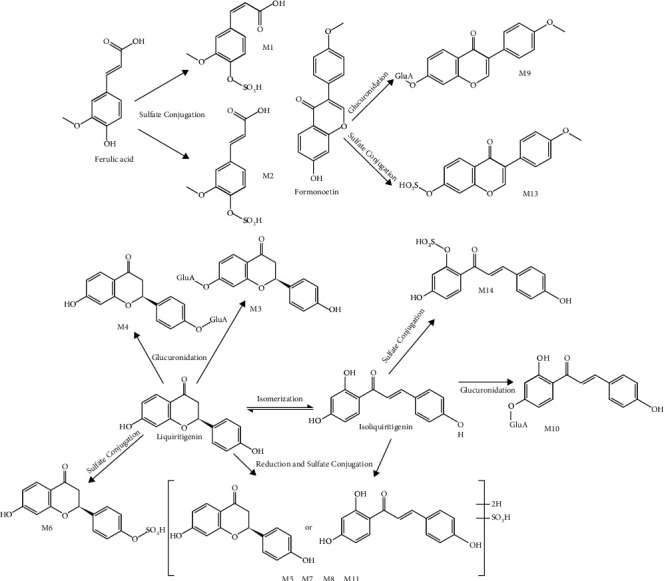
Proposed metabolic pathways of some metabolites in rat plasma after oral administration of Qi-Fu-Yin. GluA, glucuronic acid residue.

Twenty-six metabolites were preliminarily identified by comparing with data from the metabolite database, mainly including oxidation, reduction, glucuronidation, and sulfation (Table 2, Figure 5). The pathways of some metabolites are shown in Figure 6.

The [M–H]^–^ ions of M1 and M2 were at m/z 273.00, which showed a mass shift of 79.96 Da (SO_3_) from 193.05 [ferulic acid–H]^–^ and provided the fragment ions at m/z 149.02 [ferulic acid–H–CO_2_]^–^. Combined with the predicted chemical formula of C_10_H_10_O_7_S, M1 and M2 were tentatively deduced to be sulfate conjugates of ferulic acid [36] (Figure 6).

M3, M4, and M10 showed the [M–H]^–^ ion at m/z 431.10, which was 176.03 Da more than that of isoliquiritigenin. The MS_2_ spectra of M3, M4, and M10 all provided fragment ions at m/z 255.07, 175.02, and 135.01, respectively, which suggested the presence of an isoliquiritigenin group. Combining these data with the retention times [46], M3, M4, and M10 were tentatively deduced to be liquiritigenin-7-O-glucuronide, liquiritigenin-4′-O-glucuronide, and isoliquiritigenin-4′-O-glucuronide, respectively (Figure 6).

M6 and M14 showed the [M–H]^–^ ion at m/z 335.02 (C_15_H_12_O_7_S), which was 79.96 Da (SO_3_) more than that at m/z 255.07. Upon combining data from the retention time and characteristic fragmentation ions at m/z 255.07 and 135.01, M6 and M14 were identified as liquiritigenin-4′-O-sulfate and isoliquiritigenin-6′-O-sulfate, respectively (Figure 6). Similarly, the [M–H]^–^ ion of M5, M7, M8, and M11 at m/z 337.04 was approximately 2 Da more than that of M6 and M14. The product ions at m/z 257.08 were also approximately 2 Da more than those at 255.07. Combining these data with the retention time, M5, M7, M8, and M11 were deduced to be hydrogenation and sulfate conjugates of (iso)liquiritigenin (Figure 6).

M9 and M13 produced the same fragment ions at m/z 267.07, which were believed to be metabolites of formononetin; according to the adduct ions of m/z 443.0984 and 347.0230, they were identified as formononetin-7-O-glucuronide and formononetin-7-O-sulfate, respectively (Figure 6).

M12 produced fragmentation ions at m/z 207.1024 [M + H−145−H_2_O]^+^ and 189.0925 [M + H−145−2H_2_O]^+^, which suggested the presence of a phthalide group. Combining these data with the [M + H]^+^ ion at m/z 370.1316 (C_17_H_23_NO_6_S), M12 was identified as an acetylcysteine conjugate of ligustilide I or H (Table 2).

The fragment ions at m/z 459.3846, 179.0559, and 161.0453 suggested that M16 was a PPD-type ginsenoside. Combining the predicted chemical formula of C_36_H_62_O_8_ and literature [29], M15, M17-21, and M24 were identified as related metabolites of compound K, according to their retention times and chemical formulae [29] (Table 2).

M22 produced fragments of m/z 423.3243 [M + H−CO_2_]^+^ in the positive ion mode, which is in accordance with the fragmentation rules of glycyrrhetinic acid. Furthermore, M22 exhibited [M + H]^+^ at m/z 469.3312, which was determined to be C_30_H_44_O_4_; therefore, M22 was identified as the dehydrogenization of glycyrrhetinic acid. Likewise, M23 and M25 produced [M–H]^–^ ions at m/z 485.3263 and fragments of m/z 441.3357 in the negative ion mode, which represented a neutral loss of CO_2_ (44 Da), and were identified as hydroxylate conjugates of glycyrrhetinic acid (Table 2).

### 3.4. Characterization of Prototypical Components and Metabolites in the Cerebrospinal Fluid after Oral Administration of Qi-Fu-Yin

**Figure 7:**
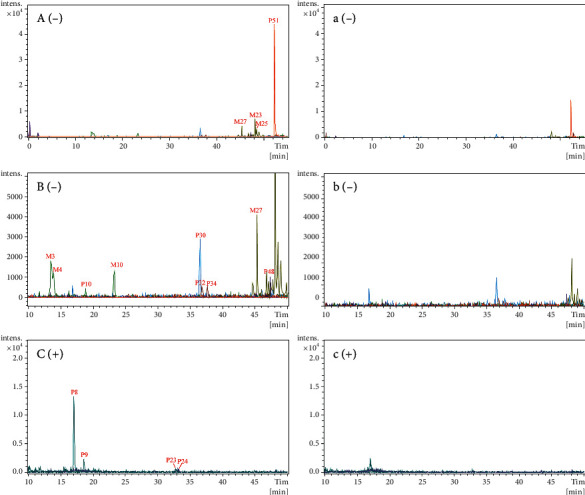
EICs of prototypical components and metabolites of Qi-Fu-Yin in the dosed and control cerebrospinal fluid in the negative and positive ion modes. (A)-(B) Dosed cerebrospinal fluid in the negative mode. (a)-(b) Control cerebrospinal fluid in the negative mode. (C) Dosed cerebrospinal fluid in the positive mode. (c) Control cerebrospinal fluid in the positive mode. Because of the presence of many metabolites in the rat cerebrospinal fluid, they cannot be displayed in the same figure and are, therefore, divided into two panels: (A) and (B).

Using the same UPLC-Q-TOF-MS conditions, 10 prototype components (P8-P10, 23, 24, 30, 32, 34, 48, and 51) and 6 metabolites (M3, 4, 10, 23, 25, and 27) were preliminarily identified by comparing the components of the drugged rat plasma, among which two components were compared with the reference standards, and others were identified by comparing the retention times, fragmentation pathways, and MS/MS spectra (Table 2 and Figure 7).

## 4. Discussion

In recent years, LC-MS technology has been widely used in the analysis of components of TCM, combining the high separation ability of liquid chromatography with the high sensitivity of mass spectrometry [47, 48]. Up to now, the only research on the identification of components in Qi-Fu-Yin was based on UPLC-Q-TOF-MS in vitro [10]. In this present study, the same 110 components were detected consistent with previous studies [10], and 70 components were preliminarily identified for the first time in vitro (Table 1, Table S1). Among them, forty-four reported components [10] were undetected, and 18 of them were lost due to different scanning ranges (Table S1).

Qi-Fu-Yin consists of seven herbs, but there is no research on the similarities and differences of components between them after decocting. For the first time, upon comparing Qi-Fu-Yin with the seven herbs, the categories of chemical components were found to be unanimous, and the number of flavonoids and organic acids in Qi-Fu-Yin was more than the sum of seven herbs; however, the opposite was true for phenylethanoid glycosides (Figure S5). Most of the chemical components could be detected in both, but 9 and 13 chemical components were only detected in the seven herbs and Qi-Fu-Yin, respectively, and the configuration of some components changed (Figure S5, Table 1). This showed that the chemical composition of Qi-Fu-Yin is not a simple addition of compounds in its single herbs.

As far as we know, the prototype components and metabolites of the seven herbs, not Qi-Fu-Yin, in the plasma after oral administration have been reported. For example, saponins in GRR [49], GRP [46], ZSS [50], flavonoids in GRP [51], ZSS [50], phthalides in ASR [36, 52], sugar esters in PRP [53], phenylethanoid glycosides, and iridoid glycoside in RRP [54] are the main components in plasma after oral administration of herbs. In this research, 51 prototypical components and 26 metabolites of Qi-Fu-Yin, including saponins, phthalides, flavonoids, sucrose esters, organic acids, alkaloids, ionones, terpene lactones, iridoid glycoside, and their derivatives have been tentatively identified in the plasma for the first time.

**Figure 8:**
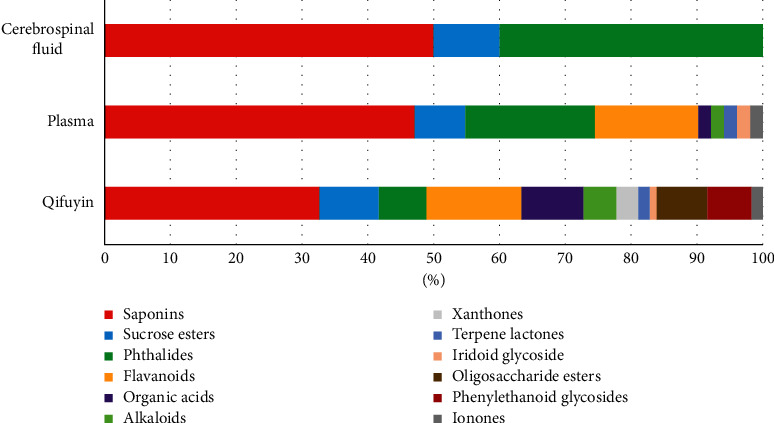
Proportion of different types of components in Qi-Fu-Yin, the plasma, and the cerebrospinal fluid.

Similarly, the prototype components and metabolites in the cerebrospinal fluid after oral administration of Qi-Fu-Yin have not been reported. Several research showed that some saponins in GRR [55, 56], GRP [57], and phthalides in ASR [58, 59] can be absorbed into the cerebrospinal fluid. In addition, saponins in GRR [60] and GRP [61], flavonoids in ZSS [62], and source esters in PRP [53] have been determined in the brain tissue homogenate. In this research, 10 prototypical components and 6 metabolites were preliminarily characterized in the rat cerebrospinal fluid after oral administration of Qi-Fu-Yin. Among them, butylidenephthalide, butylphthalide, 20(S)-ginsenoside Rh_1_, 20(R)-ginsenoside Rh_1_, zingibroside *R*_1_, and six other metabolites were detected in the cerebrospinal fluid for the first time. Some prototype components, as saponins, phthalides, and sucrose esters, could be directly absorbed into plasma and cerebrospinal fluid, and phthalides had a higher absorption rate (Figure 8). Some flavonoids, organic acids, alkaloids, xanthones, terpene lactones, and iridoid glycosides could be absorbed into the plasma, whereas other categories of chemical components were not detected in the plasma and cerebrospinal fluid.

Studies have shown that glycyrrhetinic acid [57], 3,6′-disinapoyl sucrose [63], tenuifolin [64], and senkyunolide I and H [65] can be absorbed into cerebrospinal fluid. Some components have been determined in the brain tissue homogenate [66–68], but whether these components can penetrate the BBB is unknown, and they may only exist in the astrocytes and/or vascular endothelial cells constituting the BBB. In this study, 3,6′-disinapoyl sucrose, ginsenoside Rh_1_, butylphthalide, glycyrrhetinic acid, tenuifolin, and senkyunolide I and H were detected in cerebrospinal fluid. Many studies showed that they had promising effects on neuroprotection, antiapoptosis, anti-inflammation, or antioxidative stress (Table 3). This suggested that these compounds might be potentially active components of Qi-Fu-Yin for treating AD.

## 5. Conclusions

In this study, the chemical components of Qi-Fu-Yin in the plasma and cerebrospinal fluid after oral administration of Qi-Fu-Yin were preliminarily characterized using UPLC-Q-TOF-MS. To our knowledge, this is the first systematic investigation of the metabolic profiles of the constituents of Qi-Fu-Yin. In total, 51 prototypical components and 26 metabolites were tentatively identified in plasma. The major phase I metabolic pathway of Qi-Fu-Yin involved hydrogenation and oxidation, whereas that of phase II reactions included sulfate and glucuronic acid conjugation. Furthermore, 10 prototypical components and 6 metabolites, which might be responsible for the potential activity of Qi-Fu-Yin, were preliminarily characterized in the cerebrospinal fluid. This study provides a chemical basis for elucidating the active components of Qi-Fu-Yin that play roles in the treatment of AD and should further motivate research on the mechanisms underlying the anti-AD activity of Qi-Fu-Yin.

## Figures and Tables

**Table 1:** Characterization of chemical components in Qi-Fu-Yin.

No.	*t*_*R*_ (min)	Name	Classification	Formula	Theoretical mass (Da)	Measured mass (Da)	Error (ppm)	Precursor ions	Main MS/MS fragment ions	Source	Ref.
1	0.99	Citric acid^☆^	Organic acids	C_6_H_8_O_7_	191.0197	191.0201	2.1	[M − H]^−^	129.0196, 111.009	ZSS, ASR, ARP	[16]
2	1.37	Geniposidic acid	Iridoid glycoside	C_16_H_22_O_10_	373.1140	373.1143	0.8	[M − H]^−^	211.0605, 193.0497, 167.0703, 149.0595, 123.0437	RRP	[10]
3	1.85	Decaffeoylacteoside^☆^	Phenylethanoid glycosides	C_20_H_30_O_12_	461.1664	461.1669	1.1	[M − H]^−^	375.1314, 315.1314, 297.0980, 135.0452	RRP	[17]
4	1.95	Mussaenosidic acid^☆^	Iridoid glycoside	C_16_H_24_O_10_	375.1297	375.1299	0.5	[M − H]^−^	213.0778, 169.0873, 151.0766	RRP	[18]
5	2.05	5-Caffeoylquinic acid^☆^	Organic acids	C_16_H_18_O_9_	353.0878	353.0878	0.0	[M − H]^−^	191.0563, 179.0352, 161.0245, 155.0350, 111.0088	ASR	[19]
6	2.34	3-Caffeoylquinic amide^*∗*^	Organic acids	C_16_H_19_NO_8_	354.1183	354.1178	−1.5	[M + H]^+^	192.0650, 174.0545, 146.0597		[10]
7	2.82	Ferulic acid hexoside^☆^	Organic acids	C_16_H_20_O_9_	355.1035	355.1042	2.0	[M − H]^−^	193.0509, 149.0610, 178.0271, 134.0375	ASR	[20]
8	3.01	3-Caffeoylquinic amide isomer^*∗*^	Organic acids	C_16_H_19_NO_8_	354.1183	354.1177	−1.8	[M + H]^+^	192.0650, 174.0545, 146.0597		[10]
9	3.03	Hydroxybenzoic acid^☆^	Organic acids	C_7_H_6_O_3_	137.0244	137.0244	0.0	[M − H]^−^	136.0170, 108.0215	ZSS	[16]
10	3.21	p-Hydroxybenzyl malonic acid^☆^	Organic acids	C_10_H_10_O_5_	209.0455	209.0456	0.5	[M − H]^−^	419.0982, 165.0562, 121.0662	GRP	[21]
11	3.34	Sanjoinine IB^☆^	Alkaloids	C_19_H_21_NO_4_	328.1543	328.1534	−2.7	[M + H]^+^	265.0855, 251.0665, 237.0902, 223.0712	ZSS	[22]
12	3.53	Magnocurarine^☆^	Alkaloids	C_19_H_24_NO_3_+	314.1751	314.1748	−1.0	[M]^+^	269.1179, 237.0897, 209.0947, 175.0744, 107.0491	ZSS	[22]
13	3.69	Chlorogenic acid	Organic acids	C_16_H_18_O_9_	353.0878	353.0885	2.0	[M − H]^−^	191.0563, 127.0404	ASR	[20]
14	4.26	Sibiricose A5	Sucrose esters	C_22_H_30_O_14_	517.1563	517.1568	1.0	[M − H]^−^	341.1097, 193.0512, 175.0404, 160.0169	PRP	[23]
15	4.55	4-Caffeoylquinic acid	Organic acids	C_16_H_18_O_9_	353.0878	353.0883	1.4	[M − H]^−^	191.0562, 179.0350, 173.0457, 161.0243, 111.0453, 93.0346	ASR	[10]
16	4.74	Vanillic acid	Organic acids	C_8_H_8_O_4_	167.0350	167.0351	0.6	[M − H]^−^	123.0452	ASR	[24]
17	5.32	Sibiricose A6^☆^	Sucrose esters	C_23_H_32_O_15_	547.1668	547.1678	1.8	[M − H]^−^	367.1034, 341.1094, 223.0616, 205.0508, 190.0274	PRP	[25, 26]
18	5.54	Sanjoinine K	Alkaloids	C_17_H_19_NO_3_	286.1438	286.1431	−2.3	[M + H]^+^	269.1154, 237.0905, 175.0751, 107.0492	ZSS	[16]
19	5.90	Ferulic acid hexoside isomer^☆^	Organic acids	C_16_H_20_O_9_	355.1035	355.1041	1.7	[M − H]^−^	193.0512, 149.0610, 178.0273, 134.0376	ASR	[20]
20	5.99	Darendoside B^☆^	Phenylethanoid glycosides	C_21_H_32_O_12_	475.1821	475.1830	1.9	[M − H]^−^	329.1228, 311.1144, 161.0459, 113.0247	RRP	[27]
21	6.18	Liquiritigenin-7,4′-di-O-glucoside	Flavonoids	C_27_H_32_O_14_	579.1719	579.1733	2.4	[M − H]^−^	417.1212, 255.0669, 135.0086	GRP	[28]
22	6.38	Caffeic acid	Organic acids	C_9_H_8_O_4_	179.0350	179.0352	1.1	[M − H]^−^	151.0459, 135.0499	ASR	[10]
23	7.47	Magnoflorine	Alkaloids	C_20_H_23_NO_4_+	342.1700	342.1689	−3.2	[M + H]^+^	297.1113, 282.0876, 265.0848	ZSS	[16]
24	9.46	Feruoylquinic acid^☆^	Organic acids	C_17_H_20_O_9_	367.1035	367.1043	2.2	[M − H]^−^	191.0563, 173.0461, 111.0453, 93.035	ASR	[20]
25	9.82	Lotusine^☆^	Alkaloids	C_19_H_24_NO_3_+	314.1751	314.1752	0.3	[M]^+^	269.1162, 237.0912, 209.0949, 107.0485	ZSS	[22]
26	9.94	Feruoylquinic acid isomer^☆^	Organic acids	C_17_H_20_O_9_	367.1035	367.1034	−0.3	[M − H]^−^	191.0564, 173.0457, 111.0450, 93.0347	ASR	[20]
27	10.04	Sibiricose A1	Sucrose esters	C_23_H_32_O_15_	547.1668	547.1676	1.5	[M − H]^−^	367.1040, 223.016, 190.0275	PRP	[10, 23]
28	10.23	Vicenin II	Flavonoids	C_27_H_30_O_15_	593.1512	593.1522	1.7	[M − H]^−^	503.1200, 473.1098, 383.0780, 353.0674, 325.0931,	GRP, ZSS	[16, 22]
29	10.43	Ferulic acid isomer^*∗*^^☆^	Organic acids	C_10_H_10_O_4_	193.0506	193.0506	0.0	[M − H]^−^	149.0243, 121.0298		
30	10.81	Lancerin	Xanthones	C_19_H_18_O_10_	405.0827	405.0833	1.5	[M − H]^−^	285.0410, 257.0456	PRP	[23]
31	11.30	Rehmaionoside A/B	Ionones	C_19_H_34_O_8_	435.2239	435.2246	1.6	[M + COOH]^−^	389.2223, 179.0591	RRP	[10, 17]
32	11.49	Ferulic acid	Organic acids	C_10_H_10_O_4_	193.0506	193.0507	0.5	[M − H]^−^	178.0272, 149.0609, 134.0369	ASR	[20]
33	11.97	Lancerin isomer^☆^	Xanthones	C_19_H_18_O_10_	405.0827	405.0833	1.5	[M − H]^−^	285.0413, 315.0518, 257.0458	PRP	[10]
34	12.14	Caaverine^☆^	Alkaloids	C_17_H_17_NO_2_	268.1332	268.1321	−4.1	[M − H]^−^	251.1014, 219.0829, 209.0933, 191.0862	ZSS	[22]
35	12.45	Sibiricaxanthone A/B	Xanthones	C_24_H_26_O_14_	537.1250	537.1258	1.5	[M − H]^−^	405.0832, 387.0730, 327.0524, 315.0514, 297.0412, 285.0410, 267.0303, 243.0302	PRP	[29]
36	12.45	Echinacoside	Phenylethanoid glycosides	C_35_H_46_O_20_	785.2510	785.2520	1.3	[M − H]^−^	623.2201, 461.1663, 161.0245	RRP	[30]
37	12.74	Schaftoside	Flavonoids	C_26_H_28_O_14_	563.1406	563.1408	0.4	[M − H]^−^	353.0674, 443.0992, 473.1098, 383.0778, 503.1197, 425.0877, 413.0882	GRP	[31]
38	13.12	Sibiricose A2	Sucrose esters	C_24_H_34_O_15_	561.1825	561.1832	1.2	[M − H]^−^	607.1888, 323.0991, 237.0771	PRP	[10]
39	13.41	Rehmaionoside A/B	Ionones	C_19_H_34_O_8_	435.2239	435.2239	0.0	[M + COOH]^−^	389.2223, 179.0572	RRP	[29]
40	13.79	Liquiritin	Flavonoids	C_21_H_22_O_9_	417.1191	417.1194	0.7	[M − H]^−^	255.0665, 135.0089, 119.0504	GRP	[31]
41	14.08	Polygalaxanthone III	Xanthones	C_25_H_28_O_15_	567.1355	567.1361	1.1	[M − H]^−^	447.0945, 435.0932, 417.0839, 357.0621, 345.0620, 327.0518, 315.0515, 297.0408	PRP	[10]
42	14.27	Jionoside E^☆^	Phenylethanoid glycosides	C_35_H_46_O_19_	769.2561	769.2568	0.9	[M − H]^−^	623.2197, 605.2092, 549.1662, 427.1069, 323.0996, 179.0561	RRP	[27]
43	14.37	Liquiritin apioside	Flavonoids	C_26_H_30_O_13_	549.1614	549.1616	0.4	[M − H]^−^	255.06581, 135.00719, 119.04859, 417.11804	GRP	[31]
45	14.47	Asimilobine^☆^	Alkaloids	C_17_H_17_NO_2_	268.1332	268.1324	−3.0	[M − H]^−^	251.1064, 219.0809, 201.0722, 191.0858, 179.0855	ZSS	[22]
44	14.47	Polygalaxanthone XI^☆^	Xanthones	C_25_H_28_O_15_	567.1355	567.1366	1.9	[M − H]^−^	345.0619, 315.0511	PRP	[32]
46	14.56	Jionoside A1/jionoside A2	Phenylethanoid glycosides	C_36_H_48_O_20_	799.2666	799.2672	0.8	[M − H]^−^	623.2199, 605.2092, 461.1663, 315.1110, 193.0509, 175.0403	RRP	[30]
47	14.85	Spinosin	Flavonoids	C_28_H_32_O_15_	607.1668	607.1674	1.0	[M − H]^−^	487.1252, 445.1144, 427.1039, 367.0823, 337.0722, 307.0614	ZSS	[16]
48	15.33	Swertisin	Flavonoids	C_22_H_22_O_10_	445.1140	445.1147	1.6	[M − H]^−^	355.0839, 325.0721, 297.0409	ZSS	[16]
49	15.33	Isoviolanthin/violanthin^☆^	Flavonoids	C_27_H_30_O_14_	577.1563	577.1572	1.6	[M − H]^−^	383.0777, 353.0670, 413.08783, 457.1145, 487.1248	GRP	[31]
50	15.43	Tenuifoliside B	Sucrose esters	C_30_H_36_O_17_	667.1880	667.1894	2.1	[M − H]^−^	461.1312, 205.0510, 190.0274, 137.0247, 281.0674	PRP	[23]
51	15.52	Sibiricose A4^☆^	Sucrose esters	C_34_H_42_O_19_	753.2248	753.2254	0.8	[M − H]^−^	547.1678, 529.1574, 461.1306, 367.1041, 223.0615, 205.0509, 190.0274	PRP	[29]
52	15.71	Tenuifoliside 638^☆^	Sucrose esters	C_29_H_34_O_16_	637.1774	637.1773	−0.2	[M − H]^−^	461.1309, 443.1208, 175.0402	PRP	[29]
53	16.48	Acteoside	Phenylethanoid glycosides	C_29_H_36_O_15_	623.1981	623.1989	1.3	[M − H]^−^	461.1667, 443.1555, 315.1083, 179.0349, 161.0243	RRP	[30]
54	17.06	6′′′-Vanilloylspinosin^☆^	Flavonoids	C_36_H_38_O_18_	757.1985	757.1993	1.1	[M − H]^−^	637.1556, 607.1694, 445.1143, 427.1038, 367.0827, 307.0621	ZSS	[22]
55	17.25	Senkyunolide I	Phthalides	C_12_H_16_O_4_	207.1015	207.1009	−2.9	[M + H–H_2_O]^+^	189.0822, 161.0906, 147.0752	ASR	[33]
56	17.44	Jionoside B1/Jionoside B2	Phenylethanoid glycosides	C_37_H_50_O_20_	813.2823	813.2834	1.4	[M − H]^−^	637.2359, 619.2254, 491.1780, 193.0507, 175.0402, 160.0167	RRP	[16]
57	17.63	6′′′-P-Hydroxyl-benzoyspinosin^☆^	Flavonoids	C_35_H_36_O_17_	727.1880	727.1879	−0.1	[M − H]^−^	607.1616, 445.1149, 427.1038, 325.0719, 307.0617	ZSS	[16]
58	17.73	Isoacteoside	Phenylethanoid glycosides	C_29_H_36_O_15_	623.1981	623.1990	1.4	[M − H]^−^	461.1670, 477.1405, 315.1096, 179.0351, 161.0245	RRP	[30]
59	18.69	Tenuifoliside A isomer^☆^	Sucrose esters	C_31_H_38_O_17_	681.2036	681.2044	1.2	[M − H]^−^	443.1208, 281.0672, 237.0774, 223.0616, 205.0510, 137.0246	PRP	[23]
60	18.78	Senkyunolide H	Phthalides	C_12_H_16_O_4_	207.1015	207.1008	−3.4	[M + H–H_2_O]^+^	189.0812, 161.0938, 147.0689	ASR	[33]
61	18.98	3,6′-Disinapoyl sucrose	Sucrose esters	C_34_H_42_O_19_	753.2248	753.2249	0.1	[M − H]^−^	547.1670, 529.1568, 367.1038, 265.0720, 223.0612, 205.0506, 190.0271	PRP	[10]
62	19.02	Nornuciferine^☆^	Alkaloids	C_18_H_19_NO_2_	282.1489	282.1481	−2.7	[M + H]^+^	265.1214, 250.0979, 121.0280	ZSS	[22]
63	19.05	6′′′-Sinapoyl spinosin	Flavonoids	C_39_H_42_O_19_	815.2393	815.2379	−1.7	[M + H]^+^	429.1181, 411.1037, 369.1162, 327.0855, 207.0647, 351.0833, 297.0750, 175.0385	ZSS	[16]
64	19.21	6′′′-Dihydrophaseoylspinosin^☆^	Flavonoids	C_43_H_52_O_19_	873.3176	873.3158	−2.0	[M + H]^+^	855.2986, 447.1263, 429.1140, 411.1057, 393.0969, 381.0947, 351.0846, 327.0854, 297.0752, 247.1321	ZSS	[22]
65	19.56	3,4,5-Trimethoxycinnamic acid☆	Organic acids	C_12_H_14_O_5_	237.0768	237.0770	0.8	[M − H]^−^	193.0873, 108.0215	PRP	[34]
66	19.60	6′′′-p-Coumaroyl spinosin	Flavonoids	C_37_H_38_O_17_	755.2182	755.2166	−2.1	[M + H]^+^	429.1170, 411.1080, 351.0850, 327.0854, 147.0438, 635.1770, 381.0957, 297.0750	ZSS	[16]
67	19.66	Jionoside D	Phenylethanoid glycosides	C_30_H_38_O_15_	637.2138	637.2137	−0.2	[M − H]^−^	161.0242, 461.1660, 267.0660, 175.0401	RRP	[10]
68	19.66	Arillanin A^☆^	Sucrose esters	C_33_H_40_O_18_	723.2142	723.2149	1.0	[M − H]^−^	547.1679, 265.0722, 223.0617, 205.0510, 175.0404, 160.0170	PRP	[32]
69	19.73	6′′′-Feruloyl spinosin	Flavonoids	C_38_H_40_O_18_	785.2287	785.2263	−3.1	[M + H]^+^	665.1891, 447.1275, 429.1168, 411.1068, 393.0957, 351.0852, 327.0853, 297.0750, 177.0542	ZSS	[10]
70	19.95	Tenuifoliside 652^☆^	Sucrose esters	C_30_H_36_O_16_	651.1931	651.1942	1.7	[M − H]^−^	443.1199, 281.0671, 207.0668, 175.0403, 137.0244	PRP	[29]
71	20.81	Ononin^☆^	Flavonoids	C_22_H_22_O_9_	475.1246	475.1249	0.6	[M + COOH]^−^	475.1249, 267.0664, 252.0429	GRP	[31]
72	21.00	Tenuifoliside 652 isomer^☆^	Sucrose esters	C_30_H_36_O_16_	651.1931	651.1941	1.5	[M − H]^−^	443.1199, 205.0509, 190.0272, 175.0033, 121.0297	PRP	[29]
73	21.10	Isoliquiritin apioside	Flavonoids	C_26_H_30_O_13_	549.1614	549.1620	1.1	[M − H]^−^	255.0667, 135.0090, 119.0505, 417.1200	GRP	[10]
74	21.48	Isoliquiritin	Flavonoids	C_21_H_22_O_9_	417.1191	417.1197	1.4	[M − H]^−^	255.0666, 135.0089, 119.0404	GRP	[10]
75	21.77	Leucosceptoside A	Phenylethanoid glycosides	C_30_H_38_O_15_	637.2138	637.2129	−1.4	[M − H]^−^	461.1661, 175.0400, 265.0722, 161.0239	RRP	[30]
76	21.77	Tenuifoliside A	Sucrose esters	C_31_H_38_O_17_	681.2036	681.2038	0.3	[M − H]^−^	443.1203, 281.0671, 239.0564, 179.0352, 137.0245	PRP	[10]
77	22.06	Liquiritigenin	Flavonoids	C_15_H_12_O_4_	255.0663	255.0665	0.8	[M − H]^−^	135.0086, 119.0502	GRP	[31]
78	22.64	Neoisoliquiritin^☆^	Flavonoids	C_21_H_22_O_9_	417.1191	417.1197	1.4	[M − H]^−^	255.0667, 135.0089, 119.0505	GRP	[28]
79	22.64	Notoginsenoside R1^☆^	Saponins	C_47_H_80_O_18_	977.5327	977.5334	0.7	[M + COOH]^−^	931.5284, 637.4332, 475.3809	GRR	[35]
80	22.83	6′′′-(-)-Phaseoylspinosin^☆^	Flavonoids	C_43_H_50_O_19_	869.2874	869.2884	1.2	[M − H]^−^	839.2765, 607.1683, 589.1575, 427.1045	ZSS	[22]
81	23.70	Tenuifoliose G	Oligosaccharide esters	C_66_H_84_O_38_	1483.4568	1483.4582	0.9	[M − H]^−^	1337.39795, 1295.38232, 1161.35095, 1119.34119, 997.30548, 851.27283, 753.22705, 631.18640, 452.31161, 307.08231, 175.03891, 163.03868, 145.02803	PRP	[10]
82	23.80	Senkyunolide D^☆^	Phthalides	C_12_H_14_O_4_	221.0819	221.0820	0.5	[M − H]^−^	177.0921, 147.0450	ASR	[36]
83	23.80	Tenuifoliose M	Oligosaccharide esters	C_65_H_82_O_37_	1453.4462	1453.4490	1.9	[M − H]^−^	1307.3873, 1161.3532, 997.3064, 835.2514, 307.0824, 163.0385, 145.0280	PRP	[10]
84	24.48	Ginsenoside Rg1	Saponins	C_42_H_72_O_14_	845.4904	845.4912	0.9	[M + COOH]^−^	799.4877, 637.4342, 475.3809, 161.0458, 179.0565	GRR	[31]
85	24.67	Licorice glycoside B	Flavonoids	C_35_H_36_O_15_	695.1981	695.1991	1.4	[M − H]^−^	549.1634, 163.0409, 417.1202, 255.0665, 399.1099, 531.1523, 175.0403	GRP	[31]
86	24.77	Isomartynoside^☆^	Phenylethanoid glycosides	C_31_H_40_O_15_	651.2294	651.2303	1.4	[M − H]^−^	505.1703, 475.1826, 193.0511, 175.0403, 160.017, 113.0245	RRP	[37]
87	24.77	Licorice glycoside A	Flavonoids	C_36_H_38_O_16_	725.2087	725.2095	1.1	[M − H]^−^	549.1639, 255.0668, 193.0508, 135.0086	GRP	[38]
88	24.86	Ginsenoside Re	Saponins	C_48_H_82_O_18_	991.5483	991.5501	1.8	[M + COOH]^−^	945.5442, 783.4916, 637.4329, 475.3793, 179.0562, 161.0457	GRR	[31]
89	26.11	Senkyunolide D isomer^☆^	Phthalides	C_12_H_14_O_4_	221.0819	221.0817	−0.9	[M − H]^−^	177.0920, 147.0453	ASR	[36]
90	26.31	Tenuifoliside C	Sucrose esters	C_35_H_44_O_19_	767.2404	767.2416	1.6	[M − H]^−^	529.1567, 367.1038, 237.077, 223.0613, 205.0507, 190.0271	PRP	[10]
91	26.69	Tenuifoliose T^☆^	Oligosaccharide esters	C_56_H_70_O_32_	1253.3777	1253.3792	1.2	[M − H]^−^	1223.3637, 1077.3279, 955.2908, 647.1988, 451.1232, 307.0810, 287.0549, 257.0444	PRP	[23]
92	26.88	Martynoside^☆^	Phenylethanoid glycosides	C_31_H_40_O_15_	651.2294	651.2299	0.8	[M − H]^−^	505.172, 475.1829, 193.0508, 175.0403, 160.0169, 113.0244	RRP	[17]
93	26.90	(hydroxy benzoyl)-(hydroxy cinnamoyl)-trihydroxyphenyl sucrose	Sucrose esters	C_34_H_42_O_18_	783.2353	783.2365	1.5	[M + COOH]^−^	737.2325, 615.1934, 467.1415, 323.0980, 179.0547, 161.0458, 147.0453, 121.0296	PRP	[10]
94	27.75	Tenuifoliose L	Oligosaccharide esters	C_67_H_84_O_38_	1495.4569	1495.4569	0.0	[M − H]^−^	1349.3923, 1307.3988, 163.0410, 145.0294	PRP	[10]
95	28.14	Tenuifoliose K	Oligosaccharide esters	C_57_H_70_O_32_	1265.3777	1265.3801	1.9	[M − H]^−^	1119.3395, 1077.3346, 997.3037, 163.0403, 145.0294	PRP	[10]
96	29.00	Tenuifoliose C	Oligosaccharide esters	C_58_H_72_O_33_	1295.3883	1295.3903	1.5	[M − H]^−^	1173.3653, 1119.3401, 1077.3265, 997.3061, 145.0296, 175.0404	PRP	[10]
97	29.78	Amphibine D^☆^	Alkaloids	C_36_H_49_N_5_O_5_	632.3806	632.3805	−0.2	[M + H]^+^	289.1874, 148.1111	ZSS	[16]
98	30.35	Desacylsenegasaponin B^☆^	Saponins	C_57_H_70_O_32_	1265.5808	1265.5831	1.8	[M − H]^−^	455.3179, 425.3077	PRP	[29]
99	30.44	Uralsaponin C	Saponins	C_42_H_64_O_16_	823.4122	823.4133	1.3	[M − H]^−^	647.3829, 351.0580, 193.0357	GRP	[28]
100	30.44	Tenuifoliose I	Oligosaccharide esters	C_59_H_72_O_33_	1307.3883	1307.3904	1.6	[M − H]^−^	1161.3529, 1119.3479, 1101.3331, 997.3023, 631.1891, 163.0400, 145.0299	PRP	[10]
101	30.70	Aeginetic acid^☆^	Ionones	C_15_H_24_O_4_	267.1602	267.1610	3.0	[M − H]^−^	223.1780, 205.1615, 178.9208, 153.0924	RRP	[39]
102	30.81	Methoxyl benzoyl-trimethoxyl cinnamoyl sucrose	Sucrose esters	C_32_H_40_O_17_	741.2248	741.2255	0.9	[M + COOH]^−^	237.0773, 151.0402	PRP	[10]
103	31.12	Tenuifoliose D	Oligosaccharide esters	C_60_H_74_O_34_	1337.3989	1337.4007	1.3	[M − H]^−^	1161.3546, 1119.3412, 1039.3161, 997.3030, 175.0404	PRP	[10]
104	31.41	Notoginsenoside R2	Saponins	C_41_H_70_O_13_	815.4834	815.4834	0.0	[M + COOH]^−^	769.4745, 637.4342, 475.3791, 161.0462	GRR	[40]
105	31.41	Tenuifoliose E^☆^	Oligosaccharide esters	C_58_H_72_O_33_	1295.3883	1295.3933	3.9	[M − H]^−^	1173.3506, 1119.3442, 795.2398, 175.0404, 145.0300	PRP	[29]
106	31.79	Polygalasaponin XXIII^☆^	Saponins	C_53_H_82_O_24_	1101.5123	1101.5164	3.7	[M − H]^−^	423.2925, 453.3029	PRP	[29]
107	32.08	Polygalasaponin XXVIII	Saponins	C_53_H_84_O_24_	1103.5380	1103.5328	−4.7	[M − H]^−^	455.3185, 425.3075	PRP	[23]
108	32.28	24-Hydroxyl-licorice-saponin A3	Saponins	C_48_H_72_O_22_	999.4442	999.4488	4.6	[M − H]^−^	837.3942, 351.0584, 193.0359	GRP	[10]
109	32.57	Tenuifoliose J^☆^	Oligosaccharide esters	C_59_H_72_O_33_	1307.3883	1307.3898	1.1	[M − H]^−^	1161.3549, 1039.3096, 163.0408, 145.0304	PRP	[29, 32]
110	32.81	Butylidenephthalide	Phthalides	C_12_H_12_O_2_	189.0910	189.0904	−3.2	[M + H]^+^	171.0799, 161.0954, 143.0852, 117.0694	ASR	[20]
111	32.85	Senkyunolide F^☆^	Phthalides	C_12_H_14_O_3_	205.0870	205.0880	4.9	[M − H]^−^	161.0975	ASR	[20]
112	32.92	Uralsaponin F	Saponins	C_44_H_64_O_19_	895.3969	895.3995	2.9	[M − H]^−^	719.3703, 351.0586, 193.0363	GRP	[31]
113	32.95	Onjisaponin TF	Saponins	C_59_H_94_O_28_	1249.5859	1249.5880	1.7	[M − H]^−^	1025.5362, 455.3185, 425.3077	PRP	[23]
114	33.05	Licorice saponin H2/K2^☆^	Saponins	C_42_H_62_O_16_	821.3965	821.3981	1.9	[M − H]^−^	351.0583, 193.0364, 175.0255	GRP	[28, 41]
115	33.05	22-Hydroxyl-licorice-saponin G2	Saponins	C_42_H_62_O_18_	853.3863	853.3882	2.2	[M − H]^−^	677.3568, 351.0583, 193.0365	GRP	[28]
116	33.22	Butylphthalide	Phthalides	C_12_H_14_O_2_	191.1067	191.1062	−2.6	[M + H]^+^	173.0959, 155.0842, 145.1008, 117.0698	ASR	[20]
117	33.34	Tenuifoliose B	Oligosaccharide esters	C_60_H_74_O_34_	1337.3989	1337.4027	2.8	[M − H]^−^	1161.3551, 1119.342, 1101.3324, 1039.3156, 175.0410, 145.0306	PRP	[10]
118	33.92	Ginsenoside Rf	Saponins	C_42_H_72_O_14_	845.4904	845.4928	2.8	[M + COOH]^−^	799.4880, 637.4349, 475.3820, 179.0574, 161.0466	GRR	[35]
119	33.92	Tenuifoliose H	Oligosaccharide esters	C_61_H_74_O_34_	1349.3989	1349.4019	2.2	[M − H]^−^	1307.3907, 1161.3503, 731.2194, 145.0304	PRP	[10]
120	34.40	Senkyunolide A	Phthalides	C_12_H_16_O_2_	193.1223	193.1218	−2.6	[M + H]^+^	147.1170, 175.1113, 137.0593	ASR	[20]
121	34.59	Tenuifoliose A	Oligosaccharide esters	C_62_H_76_O_35_	1379.4094	1379.4131	2.7	[M − H]^−^	1203.3649, 1161.3529, 175.041, 145.0303	PRP	[10]
122	35.08	Tenuifoliose N^☆^	Oligosaccharide esters	C_63_H_78_O_36_	1409.4200	1409.4234	2.4	[M − H]^−^	1233.3879, 175.0410	PRP	[23]
123	35.37	Ginsenoside F5^☆^	Saponins	C_41_H_70_O_13_	815.4834	815.4821	−1.6	[M + COOH]^−^	769.4765, 637.4337, 475.3807	GRR	[42]
124	35.41	Licorice saponin A3	Saponins	C_48_H_72_O_21_	983.4493	983.4518	2.5	[M − H]^−^	821.3988, 645.3687, 351.0584, 193.0366	GRP	[31]
125	35.79	24-Hydroxyl-licorice-saponin E2	Saponins	C_42_H_60_O_17_	835.3793	835.3785	−1.0	[M − H]^−^	659.3446, 351.0582, 193.0362	GRP	[28]
126	35.84	Isoliquiritigenin^*∗*^^☆^	Flavonoids	C_15_H_12_O_4_	255.0663	255.0674	4.3	[M − H]^−^	135.0094, 119.0510		[28]
127	36.04	Formononetin^*∗*^^☆^	Flavonoids	C_16_H_12_O_4_	267.0663	267.0671	3.0	[M − H]^−^	252.0458, 195.0458		[31]
128	36.32	Senkyunolide F isomer^☆^	Phthalides	C_12_H_14_O_3_	205.0870	205.0879	4.4	[M − H]^−^	161.0993	ASR	[20]
129	36.42	22*β*-Acetoxyl-glycyrrhizin	Saponins	C_44_H_64_O_18_	879.4020	879.4034	1.6	[M − H]^−^	351.0583, 193.0362	GRP	[31]
130	36.61	Tenuifolin	Saponins	C_36_H_56_O_12_	679.3699	679.3718	2.8	[M − H]^−^	455.3180, 425.3074	PRP	[10]
131	36.71	Ginsenoside F3☆	Saponins	C_41_H_70_O_13_	815.4834	815.4818	−2.0	[M + COOH]^−^	769.4761, 637.4332, 475.3810, 161.0463	GRR	[42]
132	36.90	20(S)-Ginsenoside Rh1	Saponins	C_36_H_62_O_9_	683.4376	683.4390	2.0	[M + COOH]^−^	637.4335, 475.3806, 161.0462	GRR	[10]
133	36.90	20(S)-Ginsenoside Rg2	Saponins	C_42_H_72_O_13_	829.4955	829.4969	1.7	[M + COOH]^−^	783.4911, 637.4334, 475.3807, 161.0461	GRR	[35]
134	36.90	22-Hydroxyl-glycyrrhizin	Saponins	C_42_H_62_O_17_	837.3914	837.3929	1.8	[M − H]^−^	661.3603, 485.3294, 351.0583, 193.0362	GRP	[28]
135	37.35	Senkyunolide A isomer☆	Phthalides	C_12_H_16_O_2_	193.1223	193.1217	−3.1	[M + H]^+^	147.1163, 175.1113, 137.0594	ASR	[20]
136	37.39	20(R)-Ginsenoside Rg2	Saponins	C_42_H_72_O_13_	829.4955	829.4972	2.0	[M + COOH]^−^	783.4913, 637.4332, 475.3808, 161.0462	GRR	[42]
137	37.68	20(R)-Ginsenoside Rh1	Saponins	C_36_H_62_O_9_	683.4376	683.4393	2.5	[M + COOH]^−^	637.4336, 475.3807, 161.0463	GRR	[40]
138	37.89	Jujuboside A	Saponins	C_58_H_94_O_26_	1251.6015	1251.6036	1.7	[M + COOH]^−^	1205.5983, 1073.5549, 749.4461, 455.1431, 179.0564, 161.0463	ZSS	[16]
139	38.73	Ginsenoside Rb1	Saponins	C_54_H_92_O_23_	1153.6011	1153.6033	1.9	[M + COOH]^−^	1107.5962, 945.5427, 783.4889, 621.4396, 459.3908	GRR	[31]
140	39.41	Licorice saponin E2	Saponins	C_42_H_60_O_16_	819.3809	819.3819	1.2	[M − H]^−^	645.3648, 351.0581, 193.0362	GRP	[28]
141	39.70	Ginsenoside Ro	Saponins	C_48_H_76_O_19_	955.4908	955.4918	1.0	[M − H]^−^	793.4382, 775.4275, 749.451, 731.4392, 523.3806, 455.3537, 613.3755, 569.3857, 179.0569, 119.0355	GRR	[31]
142	39.70	Ginsenoside Rc	Saponins	C_53_H_90_O_22_	1123.5906	1123.5918	1.1	[M + COOH]^−^	1077.5854, 915.5348, 459.3809, 149.0451, 191.0563	GRR	[35]
143	39.79	Licorice saponin G2	Saponins	C_42_H_62_O_17_	837.3914	837.3921	0.8	[M − H]^−^	775.3927, 661.3593, 485.3277, 351.0576, 193.0359	GRP	[28]
144	40.75	Ginsenoside Rb2	Saponins	C_53_H_90_O_22_	1123.5906	1123.5908	0.2	[M + COOH]^−^	1077.5865, 783.4945, 621.4307, 459.3789	GRR	[35]
145	41.14	Ginsenoside Rb3	Saponins	C_53_H_90_O_22_	1123.5906	1123.5907	0.1	[M + COOH]^−^	1077.5871, 783.4955, 621.4311, 459.3792	GRR	[43]
146	41.33	Rhaoglycyrrhizin	Saponins	C_48_H_72_O_20_	967.4544	967.4567	2.4	[M − H]^−^	497.1159, 321.0841, 339.0941	GRP	[10]
147	41.33	Jujuboside B	Saponins	C_52_H_84_O_21_	1045.5578	1045.5582	0.4	[M + H]^+^	733.4491, 587.39348, 533.3637, 455.3536, 437.3432, 369.2802	ZSS	[16]
148	42.59	Chikusetsusaponin IVa	Saponins	C_42_H_66_O_14_	793.4380	793.4389	1.1	[M − H]^−^	631.3854, 455.3525, 569.3834	GRR	[31]
149	42.68	Ginsenoside Rd	Saponins	C_48_H_82_O_18_	991.5483	991.5496	1.3	[M + COOH]^−^	945.5438, 783.4892, 621.438, 459.3857, 179.0563, 161.0457	GRR	[35]
150	42.78	Glycyrrhizic acid	Saponins	C_42_H_62_O_16_	821.3965	821.3972	0.9	[M − H]^−^	759.3961, 645.3648, 469.3324, 351.0572, 193.0356	GRP	[31]
151	43.19	Senkyunolide A isomer^☆^	Phthalides	C_12_H_16_O_2_	193.1223	193.1220	−1.6	[M + H]^+^	147.1166, 175.1117, 137.0599	ASR	[20]
152	44.03	6,8-Dihydroxy-1,2,4-trimethoxyxanthone^*∗*^^☆^	Xanthones	C_16_H_14_O_7_	317.0667	317.0675	2.5	[M − H]^−^	302.0444, 287.0203, 259.0254, 231.0297		[23]
153	44.61	Licorice saponin B2☆	Saponins	C_42_H_64_O_15_	807.4172	807.4178	0.7	[M − H]^−^	631.3870, 351.0572, 193.0356	GRP	[31]
154	44.62	Atractylenolide I	Terpene lactones	C_15_H_18_O_2_	231.1379	231.1373	−2.6	[M + H]^+^	213.1266, 203.1427, 189.0913, 185.1314, 157.1007	ARP	[10]
155	44.70	Atractylenolide III	Terpene lactones	C_15_H_20_O_3_	249.1485	249.1485	−0.1	[M + H]^+^	231.1405, 213.1207, 185.1277, 175.0688	ARP	[10]
156	45.19	Uralsaponin B	Saponins	C_42_H_62_O_16_	821.3965	821.3972	0.9	[M − H]^−^	759.3961, 645.3648, 469.3324, 351.0572, 193.0356	GRP	[44]
157	46.15	Licorice saponin J2	Saponins	C_42_H_64_O_16_	823.4122	823.4131	1.1	[M − H]^−^	351.0573, 193.0357	GRP	[41]
158	46.25	Ginsenoside Rg6	Saponins	C_42_H_70_O_12_	811.4849	811.4852	0.4	[M + COOH]^−^	765.4808, 619.4225, 205.0721, 161.0459	GRR	[31]
159	46.25	Senegasaponin B^☆^	Saponins	C_69_H_102_O_31_	1425.6332	1425.6381	3.4	[M − H]^−^	1395.6243, 1201.5864, 455.3163, 425.3061	PRP	[29]
160	46.25	Onjisaponin Z^☆^	Saponins	C_71_H_106_O_32_	1469.6594	1469.6600	0.4	[M − H]^−^	1245.6054, 1439.6517, 425.3061, 405.1400, 455.3165	PRP	[29]
161	46.34	Onjisaponin E	Saponins	C_71_H_106_O_33_	1485.6544	1485.6545	0.1	[M − H]^−^	455.3187, 425.3029	PRP	[23]
162	46.53	Onjisaponin Y^☆^	Saponins	C_69_H_102_O_30_	1409.6383	1409.6376	−0.5	[M − H]^−^	1379.6184, 1185.5881, 425.3062, 455.3166	PRP	[29]
163	46.53	Onjisaponin G^☆^	Saponins	C_70_H_104_O_32_	1455.6438	1455.6447	0.6	[M − H]^−^	1425.6341, 993.5078, 425.3062, 455.3166	PRP	[23]
164	46.63	Ginsenoside Rg4^☆^	Saponins	C_42_H_70_O_12_	811.4849	811.4854	0.6	[M + COOH]^−^	765.4798, 619.4212, 161.0456	GRR	[42]
165	46.82	Ginsenoside Rk3	Saponins	C_36_H_60_O_8_	665.4270	665.4271	0.2	[M + COOH]^−^	619.4211, 457.3698, 161.0458	GRR	[31]
166	46.82	Licorice saponin C2^☆^	Saponins	C_42_H_62_O_15_	805.4016	805.4020	0.5	[M − H]^−^	645.3637, 351.0575, 193.0356	GRP	[41]
167	46.92	Onjisaponin TH	Saponins	C_65_H_96_O_28_	1323.6015	1323.5991	−1.8	[M − H]^−^	455.3171, 425.3048	PRP	[23]
168	47.11	Ginsenoside Rh4	Saponins	C_36_H_60_O_8_	665.4270	665.4277	1.1	[M + COOH]^−^	619.4218, 457.3679, 161.0459	GRR	[31]
169	47.40	Zingibroside R1	Saponins	C_42_H_66_O_14_	793.4380	793.4386	0.8	[M − H]^−^	731.4390, 631.3853, 613.3751, 569.3853, 455.3538	GRR	[42]
170	47.88	Ginsenoside Rg3	Saponins	C_42_H_72_O_13_	829.4955	829.4953	−0.2	[M + COOH]^−^	783.4894, 621.4369, 459.3844, 161.0456	GRR	[31]
171	48.10	E-Ligustilide	Phthalides	C_12_H_14_O_2_	191.1067	191.1060	−3.7	[M + H]^+^	173.0959, 163.1111, 155.0845, 145.1010	ASR	[20, 33]
172	48.17	Licochalcone A^*∗*^^☆^	Flavonoids	C_21_H_22_O_4_	337.1445	337.1445	0.0	[M − H]^−^	307.0978, 281.082, 243.104		[31]
173	48.56	Isoglycyrol^*∗*^^☆^	Flavonoids	C_21_H_18_O_6_	365.1031	365.1029	−0.5	[M − H]^−^	335.0561, 307.0248, 295.0251		[31]
174	49.13	20(S)-Ginsenoside Rs3^*∗*^	Saponins	C_44_H_74_O_14_	871.5061	871.5056	−0.6	[M + COOH]^−^	825.5012, 783.4903, 621.4387, 459.3845, 765.4792		[35]
175	49.26	Atractylenolide II	Terpene lactones	C_15_H_20_O_2_	233.1536	233.1532	−1.7	[M + H]^+^	215.1431, 187.1473, 169.1047, 151.0747, 145.1009	ARP	[10]
176	49.33	20(R)-Ginsenoside Rs3^*∗*^^☆^	Saponins	C_44_H_74_O_14_	871.5061	871.5074	1.5	[M + COOH]^−^	825.5021, 783.4910, 621.4384, 459.3875, 765.4807		[35]
177	49.39	Z-Ligustilide	Phthalides	C_12_H_14_O_2_	191.1067	191.1062	−2.6	[M + H]^+^	173.0956, 163.1112, 155.0847, 145.1010	ASR	[20, 33]
178	50.00	Ginsenoside Rk1^*∗*^	Saponins	C_42_H_70_O_12_	811.4849	811.4850	0.1	[M + COOH]^−^	765.4802, 603.4275, 161.0458		[31]
179	50.19	Ginsenoside Rg5^*∗*^	Saponins	C_42_H_70_O_12_	811.4849	811.4856	0.9	[M + COOH]^−^	765.4800, 603.4263, 161.0458		[40]
180	52.21	Glycyrrhetinic acid^*∗*^^☆^	Saponins	C_30_H_46_O_4_	469.3323	469.3327	0.9	[M − H]^−^	425.3406		[31]

^*∗*^Only detected in Qi-Fu-Yin prescription, not detected in herbs; ^☆^detected in Qi-Fu-Yin prescription for the first time.

**Table 2:** Characterization of prototypical components and metabolites in rat plasma and cerebrospinal fluid after oral administration of Qi-Fu-Yin.

No.	*t*_R_ (min)	Name	Formula	Theoretical mass (Da)	Measured mass (Da)	Error (ppm)	Precursor ions	Main MS/MS fragment ions	P	CSF
P1	4.17	Sibiricose A5	C_22_H_30_O_14_	517.1563	517.1566	0.6	[M − H]^−^	193.0514, 175.0405, 160.0170	+	
P2	5.13	Sibiricose A1	C_23_H_32_O_15_	547.1668	547.1658	−1.8	[M − H]^−^	367.1030, 223.0627, 205.0508, 190.0274	+	
P3	7.31	Magnoflorine	C_20_H_23_NO_4_+	342.1700	342.1697	−0.8	[M + H]^+^	297.1119, 282.0888, 265.0843	+	
P4	13.59	Liquiritin	C_21_H_22_O_9_	417.1191	417.1189	−0.5	[M − H]^−^	255.0666, 135.0091, 119.0508	+	
P5	14.07	Polygalaxanthone III	C_25_H_28_O_15_	567.1355	567.1352	−0.5	[M − H]^−^	435.0944, 357.0600, 345.0606, 315.0522, 297.0395	+	
P6	14.16	Liquiritin apioside	C_26_H_30_O_13_	549.1614	549.1609	−0.9	[M − H]^−^	255.0662, 417.1186, 175.02373, 135.0086, 113.0248	+	
P7	14.85	Spinosin	C_28_H_32_O_15_	607.1668	607.1665	−0.5	[M − H]^−^	487.1252, 445.1177, 367.0823, 337.0722, 307.0614	+	
P8	17.24	Senkyunolide I	C_12_H_16_O_4_	207.1015	207.1012	−1.4	[M + H–H_2_O]^+^	189.0910, 161.1026, 147.0814	+	+
P9	18.77	Senkyunolide H	C_12_H_16_O_4_	207.1015	207.1013	−1.0	[M + H–H_2_O]^+^	−	+	+
P10	18.88	3,6′-Disinapoyl sucrose	C_34_H_42_O_19_	753.2248	753.2251	0.4	[M − H]^−^	547.1668, 529.1565, 265.0748, 223.0595, 205.0540	+	+
P11	19.46	3,4,5-Trimethoxycinnamic acid	C_12_H_14_O_5_	237.0768	237.0766	−0.8	[M − H]^−^	193.0870, 161.0609, 108.0217	+	
P12	20.90	Isoliquiritin apioside	C_26_H_30_O_13_	549.1614	549.1628	2.5	[M − H]^−^	255.0664, 135.0077, 119.0515	+	
P13	21.29	Isoliquiritin	C_21_H_22_O_9_	417.1191	417.1195	1.0	[M − H]^−^	255.0659, 135.0089, 119.0499	+	
P14	21.58	Tenuifoliside A	C_31_H_38_O_17_	681.2036	681.2002	−5.0	[M − H]^−^	179.0327, 137.0244	+	
P15	22.35	Liquiritigenin	C_15_H_12_O_4_	255.0663	255.0665	0.8	[M − H]^−^	135.0087, 119.0503	+	
P16	23.69	Senkyunolide D or isomer	C_12_H_14_O_4_	221.0819	221.0823	1.8	[M − H]^−^	177.0927, 147.0459	+	
P17	24.48	Ginsenoside Rg1	C_42_H_72_O_14_	845.4904	845.4900	−0.5	[M + COOH]^−^	475.3815, 179.0564, 161.0454	+	
P18	24.85	Ginsenoside Re	C_48_H_82_O_18_	991.5483	991.5436	−4.7	[M + COOH]^−^	783.4934, 475.3719, 179.0566, 161.0460	+	
P19	26.01	Senkyunolide D or isomer	C_12_H_14_O_4_	221.0819	221.0821	0.9	[M − H]^−^	177.0925, 147.0453, 134.0374	+	
P20	30.70	Aeginetic acid	C_15_H_24_O_4_	267.1602	267.1608	2.2	[M − H]^−^	178.9213, 153.0928	+	
P21	32.08	Polygalasaponin XXVIII	C_53_H_84_O_24_	1103.5280	1103.5280	0.0	[M − H]^−^	455.3189, 425.3078	+	
P22	32.66	Senkyunolide F or isomer	C_12_H_14_O_3_	205.0870	205.0872	1.0	[M − H]^−^	161.0977, 187.9911, 149.0043	+	
P23	32.90	Butylidenephthalide	C_12_H_12_O_2_	189.0910	189.0913	1.6	[M + H]^+^	171.0768, 161.0935, 143.0845, 117.0676	+	+
P24	33.22	Butylphthalide	C_12_H_14_O_2_	191.1066	191.1064	−1.0	[M + H]^+^	−	+	+
P25	34.01	Ginsenoside Rf	C_42_H_72_O_14_	845.4904	845.4887	−2.0	[M + COOH]^−^	179.0575, 161.0465	+	
P26	34.38	Senkyunolide A or isomer	C_12_H_16_O_2_	193.1223	193.1228	2.6	[M + H]^+^	147.1167, 175.1169, 137.0591	+	
P27	35.35	Licorice saponin A3	C_48_H_72_O_21_	983.4493	983.4463	−3.1	[M − H]^−^	351.0583, 193.0364	+	
P28	35.64	Isoliquiritigenin	C_15_H_12_O_4_	255.0663	255.0658	−2.0	[M − H]^−^	135.0083, 119.0498	+	
P29	35.83	Formononetin	C_16_H_12_O_4_	267.0663	267.0657	−2.2	[M − H]^−^	−	+	
P30	36.61	Tenuifolin	C_36_H_56_O_12_	679.3699	679.3718	2.8	[M − H]^−^	455.3136, 425.3101	+	+
P31	36.80	22-Hydroxyl-glycyrrhizin	C_42_H_62_O_17_	837.3914	837.3894	−2.4	[M − H]^−^	351.0584, 193.0366	+	
P32	36.89	20(S)-Ginsenoside Rh1	C_36_H_62_O_9_	683.4376	683.4367	−1.3	[M + COOH]^−^	637.4335, 475.3806, 161.0462	+	+
P33	37.37	Senkyunolide A or isomer	C_12_H_16_O_2_	193.1223	193.1224	0.5	[M + H]^+^	175.1158, 147.1162, 137.0595	+	
P34	37.66	20(R)-Ginsenoside Rh1	C_36_H_62_O_9_	683.4376	683.4367	−1.3	[M + COOH]^−^	161.0463	+	+
P35	37.85	Jujuboside A	C_58_H_94_O_26_	1251.6015	1251.5971	−3.5	[M + COOH]^−^	179.0566, 161.0465	+	
P36	38.72	Ginsenoside Rb1	C_54_H_92_O_23_	1153.6011	1153.5980	−2.7	[M + COOH]^−^	1107.5959	+	
P37	39.69	Ginsenoside Ro	C_48_H_76_O_19_	955.4908	955.4899	−0.9	[M − H]^−^	793.4379, 179.0563, 119.0352	+	
P38	39.69	Ginsenoside Rc	C_53_H_90_O_22_	1123.5906	1123.5856	−4.5	[M + COOH]^−^	459.3809, 149.0451, 191.0563	+	
P39	39.78	Licorice saponin G2	C_42_H_62_O_17_	837.3914	837.3891	−2.7	[M − H]^−^	351.056, 193.0351	+	
P40	40.75	Ginsenoside Rb2	C_53_H_90_O_22_	1123.5906	1123.5908	0.2	[M + COOH]^−^	1077.5866	+	
P41	41.32	Rhaoglycyrrhizin	C_48_H_72_O_20_	967.4544	967.4506	−3.9	[M − H]^−^	1077.5859	+	
P42	42.76	Glycyrrhizic acid	C_42_H_62_O_16_	821.3965	821.3942	−2.8	[M − H]^−^	645.3641, 351.0564, 193.0351, 175.0249	+	
P43	42.76	Ginsenoside Rd	C_48_H_82_O_18_	991.5483	991.5474	−0.9	[M + COOH]^−^	179.0564, 161.0456	+	
P44	44.63	Atractylenolide I	C_15_H_18_O_2_	231.1379	231.1378	−0.4	[M + H]^+^	−	+	
P45	46.13	Licorice saponin J2	C_42_H_64_O_16_	823.4122	823.4091	−3.8	[M − H]^−^	351.0573, 193.0357	+	
P46	46.90	Ginsenoside Rk3	C_36_H_60_O_8_	665.4270	665.4248	−3.3	[M + COOH]^−^	161.0449	+	
P47	47.09	Ginsenoside Rh4	C_36_H_60_O_8_	665.4270	665.4258	−1.8	[M + COOH]^−^	161.0450	+	
P48	47.38	Zingibroside R1	C_42_H_66_O_14_	793.4380	793.4374	−0.8	[M − H]^−^	731.4388, 631.3849	+	+
P49	47.86	Ginsenoside Rg3	C_42_H_72_O_13_	829.4955	829.4934	−2.5	[M − H]^−^	783.4886, 621.4365, 459.3812, 161.0454	+	
P50	49.40	*Z*-Ligustilide	C_12_H_14_O_2_	191.1066	191.1070	2.1	[M + H]^+^	−	+	
P51	52.30	Glycyrrhetinic acid	C_30_H_46_O_4_	469.3323	469.3316	−1.5	[M − H]^−^	425.3414	+	+
M1	8.78	Ferulic acid-4-sulfate	C_10_H_10_O_7_S	273.0074	273.0074	0.0	[M − H]^−^	193.0507, 149.0246	+	
M2	9.45	Ferulic acid-4-sulfate isomer	C_10_H_10_O_7_S	273.0074	273.0073	−0.4	[M − H]^−^	193.0504, 149.0245	+	
M3	13.59	Liquiritigenin-7-O-glucuronide	C_21_H_20_O_10_	431.0984	431.0977	−1.6	[M − H]^−^	255.0662, 175.0250, 135.0088	+	+
M4	13.97	Liquiritigenin-4′-O-glucuronide	C_21_H_20_O_10_	431.0984	431.0982	−0.5	[M − H]^−^	255.0662, 175.025, 135.0088	+	+
M5	15.70	Liquiritigenin+2H + sulfate	C_15_H_14_O_7_S	337.0382	337.0380	−0.6	[M − H]^−^	257.0824	+	
M6	17.83	Liquiritigenin-4′-O-sulfate	C_15_H_12_O_7_S	335.0231	335.0225	−1.8	[M − H]^−^	255.0664, 135.0088, 119.0503	+	
M7	19.36	(Iso)Liquiritigenin+2H + sulfate	C_15_H_14_O_7_S	337.0382	337.0383	0.3	[M − H]^−^	257.0823, 151.0401	+	
M8	20.81	(Iso)Liquiritigenin+2H + sulfate	C_15_H_14_O_7_S	337.0382	337.0385	0.9	[M − H]^−^	257.0820, 151.0398	+	
M9	21.10	Formononetin-7-O-glucuronide	C_22_H_20_O_10_	443.0984	443.0984	0.0	[M − H]^−^	267.0661, 175.0249, 135.0453	+	
M10	23.12	Isoliquiritigenin-4′-O-glucuronide	C_21_H_20_O_10_	431.0984	431.0978	−1.4	[M − H]^−^	255.0662, 175.0247, 135.0088	+	+
M11	27.07	Isoliquiritigenin+2H + sulfate	C_15_H_14_O_7_S	337.0382	337.0390	2.4	[M − H]^−^	257.0821	+	
M12	28.20	Acetylcysteine conjugate of senkyunolide I or senkyunolide H	C_17_H_23_NO_6_S	370.1324	370.1316	−2.2	[M + H]^+^	207.1024, 189.0925, 161.0957	+	
M13	29.38	Formononetin-7-O-sulfate	C_16_H_12_O_7_S	347.0231	347.0230	−0.3	[M − H]^−^	267.0664, 252.0429	+	
M14	29.67	Isoliquiritigenin-6′-O-sulfate	C_15_H_12_O_7_S	335.0231	335.0236	1.5	[M − H]^−^	255.0666, 135.009, 119.0508	+	
M15	38.72	Compound K-H2	C_36_H_60_O_8_	619.4215	619.4193	−3.6	[M − H]^−^	457.3683, 439.3216	+	
M16	45.27	Compound K	C_36_H_62_O_8_	621.4372	621.4355	−2.7	[M − H]^−^	459.3846, 179.0559, 161.0453	+	
M17	45.94	Compound K+2O-2H2	C_36_H_58_O_10_	665.3906	665.3883	−3.5	[M − H]^−^	651.4118, 409.2751, 375.2533	+	
M18	46.42	Compound K+3O-H2	C_36_H_59_O_11_	667.4063	667.4047	−2.4	[M − H]^−^	605.4042, 491.3720, 175.0237, 113.0242	+	
M19	46.90	Compound K+3O-H2	C_36_H_59_O_11_	667.4063	667.4042	−3.1	[M − H]^−^	605.4029, 491.3724, 175.0241, 113.0242	+	
M20	46.99	Compound K+2O-2H2	C_36_H_58_O_10_	665.3906	665.3893	−2.0	[M − H]^−^	651.4113, 409.2746, 375.2527	+	
M21	47.76	Compound K+2O-2H2	C_36_H_58_O_10_	665.3906	665.3897	−1.4	[M − H]^−^	651.4119, 409.2752, 375.2535	+	
M22	48.13	Glycyrrhetinic acid-2H	C_30_H_44_O_4_	469.3318	469.3312	−1.3	[M+H]^+^	451.3203, 423.3243	+	
M23	48.15	Glycyrrhetinic acid + O	C_30_H_46_O_5_	485.3272	485.3263	−1.9	[M − H]^−^	441.3357	+	+
M24	48.34	Compound K+2O-2H2	C_36_H_58_O_10_	665.3906	665.3904	−0.3	[M − H]^−^	491.3368, 473.3269, 443.3161, 193.0352, 175.0246, 113.0242	+	
M25	48.92	Glycyrrhetinic acid + O	C_30_H_46_O_5_	485.3272	485.3256	−3.3	[M − H]^−^	441.3361	+	+
M26	49.59	Protopanaxadiol+2O + H2	C_30_H_50_O_5_	489.3585	489.3575	−2.0	[M − H]^−^	473.3261, 445.3677, 375.2896	+	
M27	45.36	Glycyrrhetinic acid + O	C_30_H_46_O_5_	485.3272	485.3279	1.4	[M − H]^−^	441.3383		+

P, plasma; CSF, cerebrospinal fluid; −, not detected +, detected.

**Table 3:** Effects of prototype components in the cerebrospinal fluid after oral administration of Qi-Fu-Yin anti-Alzheimer's disease.

Compound	Samples	Biomarkers	Effects	References
3,6′-Disinapoyl sucrose	Glutamate and H_2_O_2_-induced SHSY5Y cells	Protein expression of CREB↑ Protein expression of BDNF↑	Neuroprotection	[69]
	Glutamate-induced SHSY5Y cells	mRNA expression of Bax↓ mRNA expression of Bcl-2↑	Antiapoptosis	[70]
Ginsenoside Rh1	Mice (6-month-old)	Number of crosses, time spent in platform quadrant↑ in the Morris water maze test Protein expression of BDNF↑	Neuroprotection	[71]
	IFN-*γ*-stimulated BV2 cells	Amounts of NO, ROS, and TNF-*α*↓	Anti-inflammation	[72]
	Scopolamine-induced amnesic mice	Escape latency↓ in the Morris water maze test Activity of SOD and CAT↑	Antioxidative stress	[73]
Butylphthalide	APP/PS1 mice	Escape latency↓, the time spent and travel distance in the target quadrant↑ in the Morris water maze test	Neuroprotection	[74]
	A*β*_1-42_-induced SD rats	Protein expression of MAPK↓	Antiapoptosis	[75]
Senkyunolide H	1-Methyl-4-phenylpyridinium-induced PC12 cells	Amounts of ROS, MDA↓ Activities of SOD, CAT, GSH-Px↑	Antioxidative stress	[76]
		Protein expression of Bax and caspase-3↓	Antiapoptosis	[76]
Tenuifolin	A*β*_1-42_-induced BV2 cells	Amounts of TNF-*α*, IL-6, and IL-1*β*↓	Anti-inflammation	[77]
		mRNA expression of iNOS and COX-2↓ Amount of NO↓	Antioxidative stress	[77]
Senkyunolide I	Glutamate-induced Neuro2a cells	Amount of caspase-3↓	Antiapoptosis	[78]
Glycyrrhetinic acid	BACE1 FRET assay	Activity of BACE1↓	Neuroprotection	[79]

↓, decrease; ↑, increase; A*β*, amyloid-*β*; CREB, cyclic AMP response element binding protein; BDNF, brain-derived neurotrophic factor; Bax, Bcl-2 associated X protein; Bcl-2, B cell lymphoma/leukemia-2; NO, nitric oxide; ROS, reactive oxygen species; TNF-*α*, tumor necrosis factor-*α*; MDA, malondialdehyde; SOD, superoxide dismutase; CAT, catalase; GSH-Px, glutathione peroxidase; IL-6, interleukin 6; IL-1*β*, interleukin 1*β;* iNOS, inducible nitric oxide synthase; COX-2, cyclooxygenase-2; MAPK, mitogen-activated protein kinase; BACE1: *β*-site APP cleaving enzyme 1.

## Data Availability

The data used to support the findings of this study are included within the article and are available from the corresponding author upon request.

## Supplementary Materials

Supplementary Materials Figure S1. Base peak chromatograms of Qi-Fu-Yin and seven herbs in the positive (+) and negative (−) ion modes. QFY, Qi-Fu-Yin; GRR, Ginseng Radix et Rhizoma; RRP, Rehmanniae Radix Preparata; ASR, Angelicae Sinensis Radix; ARP, Atractylodis Macrocephala Rhizoma Preparata; GRP, Glycyrrhizae Radix et Rhizoma Preparata Cum Melle; ZSS, Ziziphi Spinosae Semen; PRP, Polygalae Radix Preparata. Figure S2. MS/MS spectra and the proposed fragmentation pathways of acteoside, schaftoside, and spinosyn. (A) MS/MS spectra and the proposed fragmentation pathways for acteoside. (B) MS/MS spectra and the proposed fragmentation pathways of schaftoside. (C) MS/MS spectra and the proposed fragmentation pathways for spinosin. Figure S3. MS/MS spectra and the proposed fragmentation pathways of tenuifoliside C. Figure S4. Extracted ion chromatograms of senkyunolide I and H in the dosed and control plasma in the negative ion mode. Figure S5. Difference between the chemical components or category and number of chemical components of Qi-Fu-Yin and the seven herbs. (A) Difference between the chemical components of Qi-Fu-Yin and the seven herbs. (B) Difference between the category and number of chemical components of Qi-Fu-Yin and the seven herbs. Table S1. Comparison between the current study and Li's study.
