# Supplementary material for: Identification of Chemical Components of Qi-Fu-Yin and Its Prototype Components and Metabolites in Rat Plasma and Cerebrospinal Fluid via UPLC-Q-TOF-MS
Source: Evid Based Complement Alternat Med. 2021 Dec 28;2021:1995766. doi: 10.1155/2021/1995766 (PMC8727097; doi:10.1155/2021/1995766)
Supplement: Supplementary Materials — Figure S1. Base peak chromatograms of Qi-Fu-Yin and seven herbs in the positive (+) and negative (−) ion modes. QFY, Qi-Fu-Yin; GRR, Ginseng Radix et Rhizoma; RRP, Rehmanniae Radix Preparata; ASR, Angelicae Sinensis Radix; ARP, Atractylodis Macrocephala Rhizoma Preparata; GRP, Glycyrrhizae Radix et Rhizoma Preparata Cum Melle; ZSS, Ziziphi Spinosae Semen; PRP, Polygalae Radix Preparata. Figure S2. MS/MS spectra and the proposed fragmentation pathways of acteoside, schaftoside, and spinosyn. (A) MS/MS spectra and the proposed fragmentation pathways for acteoside. (B) MS/MS spectra and the proposed fragmentation pathways of schaftoside. (C) MS/MS spectra and the proposed fragmentation pathways for spinosin. Figure S3. MS/MS spectra and the proposed fragmentation pathways of tenuifoliside C. Figure S4. Extracted ion chromatograms of senkyunolide I and H in the dosed and control plasma in the negative ion mode. Figure S5. Difference between the chemical components or category and number of chemical components of Qi-Fu-Yin and the seven herbs. (A) Difference between the chemical components of Qi-Fu-Yin and the seven herbs. (B) Difference between the category and number of chemical components of Qi-Fu-Yin and the seven herbs. Table S1. Comparison between the current study and Li's study. [file 1995766.f1.zip › 1995766.f1/Supplementary Table.docx]

| Supplementary Table 1 Comparison between this study and Li's report | | | | | | | |
| --- | --- | --- | --- | --- | --- | --- | --- |
| Common components | Classification | Common components | Classification | Reduced components | Classification | Added components | Classification |
| Rehmaionoside A/Rehmaionoside B | ionone | Polygalaxanthone III | xanthones | Norisoboldine | alkaloids | Senkyunolide A | phthalides |
| Rehmaionoside A/B | ionone | 3-caffeoylquinic amide | organic acids | Zizyphusine | alkaloids | Senkyunolide A isomer | phthalides |
| Sibiricose A5 | sucrose esters | Chlorogenic acid | organic acids | 6-C-glucopyransoyl-8-Carabinopyransoyl-5,7dihydroxyflavanone | flavonoids | Senkyunolide D | phthalides |
| Sibiricose A1 | sucrose esters | 4-caffeoylquinic acid | organic acids | Isoschaftoside | flavonoids | Senkyunolide D isomer | phthalides |
| Sibiricose A2 | sucrose esters | Vanillic acid | organic acids | Naringenine-4'-Orhamnosidoglucoside | flavonoids | Senkyunolide F | phthalides |
| Tenuifoliside B | sucrose esters | Caffeic acid | organic acids | 3,4-dicaffeoylquinic acid | organic acids | Senkyunolide F isomer | phthalides |
| Tenuifoliside A | sucrose esters | Ferulic acid | organic acids | 3,5-dicaffeoylquinic acid | organic acids | Darendoside B | phenylethanoid glycosides |
| Tenuifoliside C | sucrose esters | Chlorogenic acid | organic acids | Naringin | flavonoids | decaffeoylacteoside | phenylethanoid glycosides |
| (hydroxy benzoyl)-(hydroxy cinnamoyl)-trihydroxyphenyl sucrose | sucrose esters | Atractylenolide I | terpene lactones | 4,5-dicaffeoylquinic acid | organic acids | Martynoside | phenylethanoid glycosides |
| methoxyl benzoyl-trimethoxyl cinnamoyl sucrose | sucrose esters | Atractylenolide III | terpene lactones | Hesperidin | flavonoids | Jionoside E | phenylethanoid glycosides |
| Ginsenoside Rg1 | saponins | Atractylenolide II | terpene lactones | Sanjoinine Ia (pronuciferin) | alkaloids | Isomartynoside | phenylethanoid glycosides |
| Ginsenoside Re | saponins | Sanjoinine K | alkaloids | Sanjoinine E (nuciferin) | alkaloids | Tenuifoliose E | oligosaccharide esters |
| Uralsaponin C | saponins | Magnoflorine | alkaloids | Apigenin-7-O-glucuronide | flavonoids | Tenuifoliose J | oligosaccharide esters |
| 24-hydroxyl-licorice-saponin A3 | saponins | Sanjoinine Ib | alkaloids | Sanjoinine F | alkaloids | Tenuifoliose N | oligosaccharide esters |
| Polygalasaponin XXVIII | saponins | Liquiritigenin-7,4'-di-O-glucoside | flavonoids | Tenuifoliose S | oligosaccharide esters | Tenuifoliose T | oligosaccharide esters |
| Uralsaponin F | saponins | Vicenin II | flavonoids | Tenuifoliose F | oligosaccharide esters | Mussaenosidic acid | iridoid glycoside |
| Onjisaponin TF | saponins | Schaftoside | flavonoids | Tenuifoliose P | oligosaccharide esters | 6'''-(-)-Phaseoylspinosin | flavonoids |
| 22-hydroxyl-licorice-saponin G2 | saponins | Liquiritin | flavonoids | Apigenin-7-O-methylglucuronide | flavonoids | 6'''-P-hydroxyl-benzoyspinosin | flavonoids |
| Licorice-saponin A3 | saponins | Liquiritin apioside | flavonoids | Sanjoinine A | alkaloids | 6'''-Dihydrophaseoylspinosin | flavonoids |
| Ginsenoside Rf | saponins | Spinosin | flavonoids | Tenuifoliose O | oligosaccharide esters | 6'''-vanilloylspinosin | flavonoids |
| Notoginsenoside R2 | saponins | Swertisin | flavonoids | Uralsaponin D | saponins | Isoglycyrol | flavonoids |
| 22-hydroxyl-glycyrrhizin | saponins | 6'''-sinapoyl spinosin | flavonoids | Onjisaponin TG | saponins | IsoLiquiritigenin | flavonoids |
| Tenuifolin | saponins | 3,6'-disinapoyl sucrose | flavonoids | Uralsaponin A | saponins | Isoviolanthin /Violanthin | flavonoids |
| 20(S)-ginsenoside Rh1 | saponins | 6'''-p-coumaroyl spinosin | flavonoids | Onjisaponin TE | saponins | Licochalcone A | flavonoids |
| 20(S)-ginsenoside Rg2 | saponins | 6'''-feruloyl spinosin | flavonoids | z-butylidenephthalide | phthalides | Neoisoliquiritin | flavonoids |
| 20(R)-Ginsenoside Rh1 | saponins | Isoliquiritin apioside | flavonoids | 22-acetoxyl-glycyrrhaldehyde | saponins | Ononin | flavonoids |
| 20(R)-ginsenoside Rg2 | saponins | Isoliquiritin | flavonoids | Onjisaponin V | saponins | Formononetin | flavonoids |
| Jujuboside A | saponins | Liquiritigenin | flavonoids | Onjisaponin S | saponins | Amphibine D | alkaloids |
| Ginsenoside Rb1 | saponins | Licorice-glycoside B | flavonoids | Onjisaponin Sg | saponins | Asimilobine | alkaloids |
| Ginsenoside Rc | saponins | Licorice-glycoside A | flavonoids | 22-dehydroxyl-uralsaponin C | saponins | Caaverine | alkaloids |
| Ginsenoside Ro | saponins | Geniposidic acid | iridoid glycoside | Senegin IV | saponins | Lotusine | alkaloids |
| Licorice-saponin E2 | saponins | Tenuifoliose G | oligosaccharide esters | Onjisaponin O | saponins | Nornuciferine | alkaloids |
| Ginsenoside Rb2 | saponins | Tenuifoliose M | oligosaccharide esters | Onjisaponin L | saponins | Magnocurarine | alkaloids |
| Licorice-saponin G2 | saponins | Tenuifoliose L | oligosaccharide esters | Onjisaponin Fg | saponins | 3,4,5-trimethoxycinnamic acid | organic acids |
| Ginsenoside Rb3 | saponins | Tenuifoliose K | oligosaccharide esters | Onjisaponin R | saponins | 3-caffeoylquinic amide isomer | organic acids |
| Rhaoglycyrrhizin | saponins | Tenuifoliose C | oligosaccharide esters | Senegin III | saponins | 5-caffeoylquinic acid | organic acids |
| Jujuboside B | saponins | Tenuifoliose I | oligosaccharide esters | Onjisaponin F | saponins | Citric Acid | organic acids |
| Ginsenoside Rd | saponins | Tenuifoliose D | oligosaccharide esters | Onjisaponin Vg | saponins | Ferulic acid hexoside | organic acids |
| Chikusetsusaponin IVa | saponins | Tenuifoliose B | oligosaccharide esters | Polygalasaponin XXXII | saponins | Ferulic acid hexoside isomer | organic acids |
| Glycyrrhizin | saponins | Tenuifoliose H | oligosaccharide esters | Onjisaponin J | saponins | Ferulic acid isomer | organic acids |
| Uralsaponin B | saponins | Tenuifoliose A | oligosaccharide esters | Onjisaponin Ng | saponins | Feruoylquinic acid | organic acids |
| Licorice-saponin J2 | saponins | Echinacoside | phenylethanoid glycosides | Onjisaponin W | saponins | Feruoylquinic acid isomer | organic acids |
| Onjisaponin E | saponins | Jionoside A1/Jionoside A2 | phenylethanoid glycosides | Onjisaponin Gg | saponins | Hydroxybenzoic acid | organic acids |
| Ginsenoside Rk3 | saponins | Acteoside | phenylethanoid glycosides | isomer of senegin IV | saponins | p-Hydroxybenzyl malonic acid | organic acids |
| Ginsenoside Rh4 | saponins | Jionoside B1/Jionoside B2 | phenylethanoid glycosides | The 2 columns represent 44 reduced components  and their classifications | | 6,8-Dihydroxy-1,2,4-trimethoxyxanthone | xanthones |
| Onjisaponin TH | saponins | Isoacteoside | phenylethanoid glycosides | Yellow means the molecular weight is more than 1500Da, which which is beyond our scanning range. | | Lancerin | xanthones |
| Zingibroside R1 | saponins | Jionoside D | phenylethanoid  glycosides | |  | Polygalaxanthone XI | xanthones |
| Ginsenoside Rg3 | saponins | Leucosceptoside A | phenylethanoid  glycosides | |  | 24-hydroxyl-licorice-saponin E2 | saponins |
| Ginsenoside Rs3 | saponins | Senkyunolide H/Senkyunolide I | phthalides |  |  | Desacylsenegasaponin B | saponins |
| Ginsenoside Rk1 | saponins | Senkyunolide H/Senkyunolide I | phthalides |  |  | Ginsenoside F3 | saponins |
| Ginsenoside Rg5 | saponins | E-butylidenephthalide | phthalides |  |  | Ginsenoside F5 | saponins |
| 22-acetoxyl-glycyrrhizin | saponins | Senkyunolide A | phthalides |  |  | Ginsenoside Rg4 | saponins |
| Ginsenoside Rg6 | saponins | Butylphthalide | phthalides |  |  | Ginsenoside Rs3 | saponins |
| Lancerin | xanthones | Z-ligustilide | phthalides |  |  | Glycyrrhetinic acid | saponins |
| Sibiricaxanthone A | xanthones | E-ligustilide | phthalides |  |  | Licorice saponin B2 | saponins |
| The 4 columns represent 110 common components and their classifications | | | |  |  | Licorice saponin C2 | saponins |
|  |  |  |  |  |  | Licorice saponin H2/K2 | saponins |
|  |  |  |  |  |  | Onjisaponin G | saponins |
|  |  |  |  |  |  | Onjisaponin Y | saponins |
|  |  |  |  |  |  | Onjisaponin Z | saponins |
|  |  |  |  |  |  | Polygalasaponin XXIII | saponins |
|  |  |  |  |  |  | Senegasaponin B | saponins |
|  |  |  |  |  |  | Notoginsenoside R1 | saponins |
|  |  |  |  |  |  | Arillanin A | sucrose esters |
|  |  |  |  |  |  | Sibiricose A4 | sucrose esters |
|  |  |  |  |  |  | Tenuifoliside 638 | sucrose esters |
|  |  |  |  |  |  | Tenuifoliside 652 | sucrose esters |
|  |  |  |  |  |  | Tenuifoliside 652 | sucrose esters |
|  |  |  |  |  |  | Tenuifoliside A | sucrose esters |
|  |  |  |  |  |  | Aeginetic acid | ionone |
|  |  |  |  |  |  | The 2 columns represent 70 new components | |
