# Supplementary figures and images for: Identification of Chemical Components of Qi-Fu-Yin and Its Prototype Components and Metabolites in Rat Plasma and Cerebrospinal Fluid via UPLC-Q-TOF-MS
Source: Evid Based Complement Alternat Med. 2021 Dec 28;2021:1995766. doi: 10.1155/2021/1995766 (PMC8727097; doi:10.1155/2021/1995766)

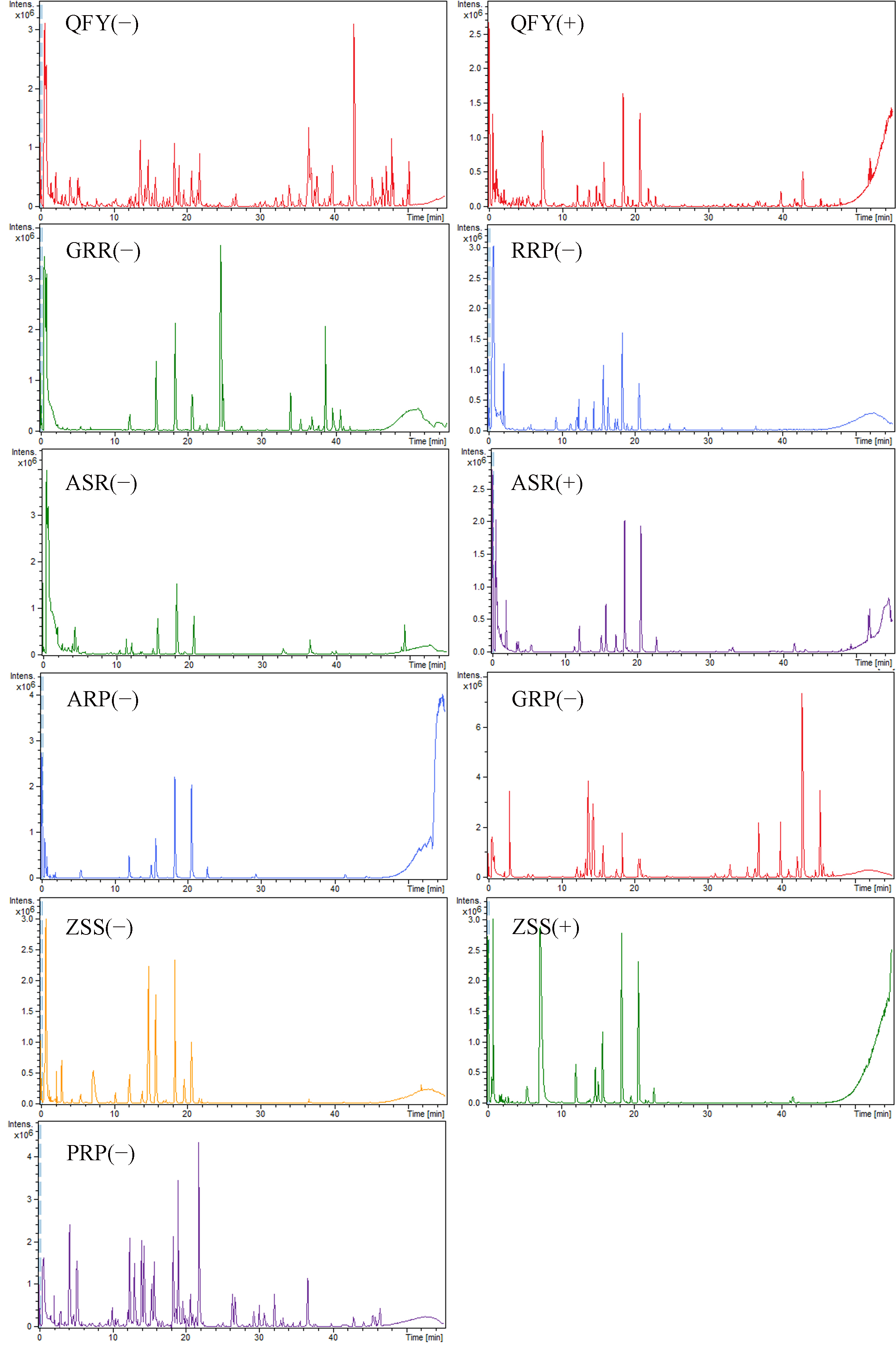

Supplement: Supplementary Materials — Figure S1. Base peak chromatograms of Qi-Fu-Yin and seven herbs in the positive (+) and negative (−) ion modes. QFY, Qi-Fu-Yin; GRR, Ginseng Radix et Rhizoma; RRP, Rehmanniae Radix Preparata; ASR, Angelicae Sinensis Radix; ARP, Atractylodis Macrocephala Rhizoma Preparata; GRP, Glycyrrhizae Radix et Rhizoma Preparata Cum Melle; ZSS, Ziziphi Spinosae Semen; PRP, Polygalae Radix Preparata. Figure S2. MS/MS spectra and the proposed fragmentation pathways of acteoside, schaftoside, and spinosyn. (A) MS/MS spectra and the proposed fragmentation pathways for acteoside. (B) MS/MS spectra and the proposed fragmentation pathways of schaftoside. (C) MS/MS spectra and the proposed fragmentation pathways for spinosin. Figure S3. MS/MS spectra and the proposed fragmentation pathways of tenuifoliside C. Figure S4. Extracted ion chromatograms of senkyunolide I and H in the dosed and control plasma in the negative ion mode. Figure S5. Difference between the chemical components or category and number of chemical components of Qi-Fu-Yin and the seven herbs. (A) Difference between the chemical components of Qi-Fu-Yin and the seven herbs. (B) Difference between the category and number of chemical components of Qi-Fu-Yin and the seven herbs. Table S1. Comparison between the current study and Li's study. [file 1995766.f1.zip › 1995766.f1/Supplementary Fig 1.jpg]

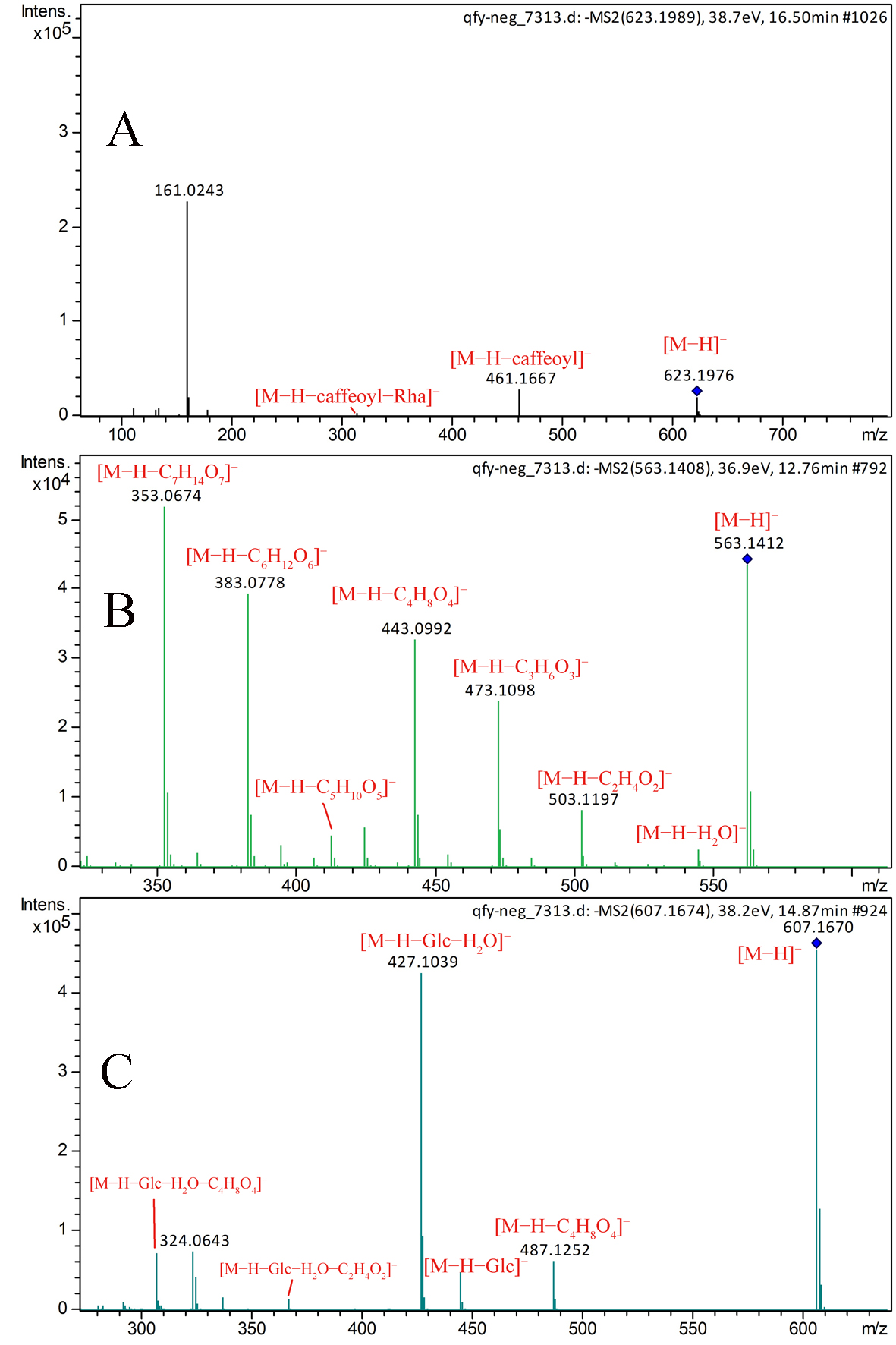

Supplement: Supplementary Materials — Figure S1. Base peak chromatograms of Qi-Fu-Yin and seven herbs in the positive (+) and negative (−) ion modes. QFY, Qi-Fu-Yin; GRR, Ginseng Radix et Rhizoma; RRP, Rehmanniae Radix Preparata; ASR, Angelicae Sinensis Radix; ARP, Atractylodis Macrocephala Rhizoma Preparata; GRP, Glycyrrhizae Radix et Rhizoma Preparata Cum Melle; ZSS, Ziziphi Spinosae Semen; PRP, Polygalae Radix Preparata. Figure S2. MS/MS spectra and the proposed fragmentation pathways of acteoside, schaftoside, and spinosyn. (A) MS/MS spectra and the proposed fragmentation pathways for acteoside. (B) MS/MS spectra and the proposed fragmentation pathways of schaftoside. (C) MS/MS spectra and the proposed fragmentation pathways for spinosin. Figure S3. MS/MS spectra and the proposed fragmentation pathways of tenuifoliside C. Figure S4. Extracted ion chromatograms of senkyunolide I and H in the dosed and control plasma in the negative ion mode. Figure S5. Difference between the chemical components or category and number of chemical components of Qi-Fu-Yin and the seven herbs. (A) Difference between the chemical components of Qi-Fu-Yin and the seven herbs. (B) Difference between the category and number of chemical components of Qi-Fu-Yin and the seven herbs. Table S1. Comparison between the current study and Li's study. [file 1995766.f1.zip › 1995766.f1/Supplementary Fig 2.jpg]

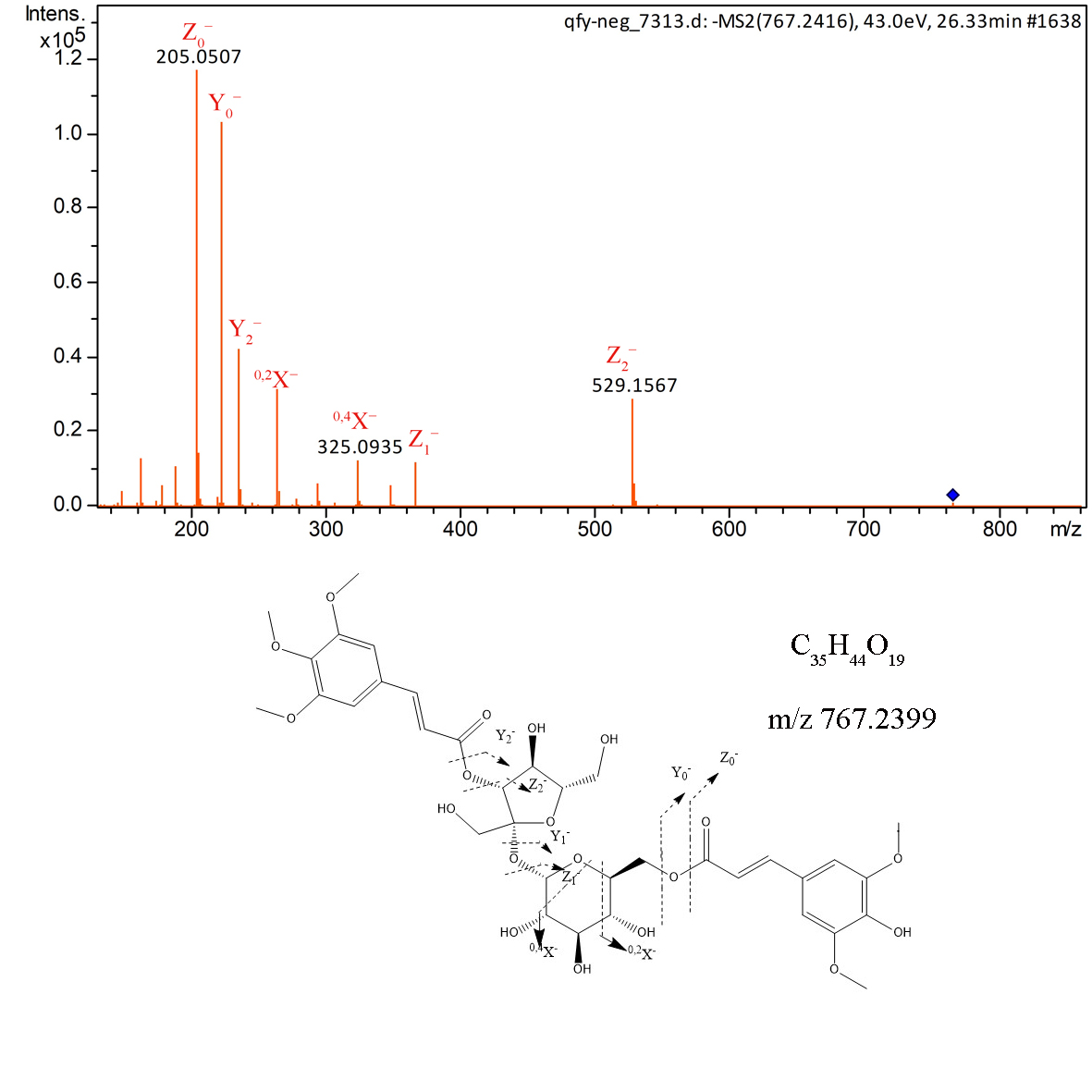

Supplement: Supplementary Materials — Figure S1. Base peak chromatograms of Qi-Fu-Yin and seven herbs in the positive (+) and negative (−) ion modes. QFY, Qi-Fu-Yin; GRR, Ginseng Radix et Rhizoma; RRP, Rehmanniae Radix Preparata; ASR, Angelicae Sinensis Radix; ARP, Atractylodis Macrocephala Rhizoma Preparata; GRP, Glycyrrhizae Radix et Rhizoma Preparata Cum Melle; ZSS, Ziziphi Spinosae Semen; PRP, Polygalae Radix Preparata. Figure S2. MS/MS spectra and the proposed fragmentation pathways of acteoside, schaftoside, and spinosyn. (A) MS/MS spectra and the proposed fragmentation pathways for acteoside. (B) MS/MS spectra and the proposed fragmentation pathways of schaftoside. (C) MS/MS spectra and the proposed fragmentation pathways for spinosin. Figure S3. MS/MS spectra and the proposed fragmentation pathways of tenuifoliside C. Figure S4. Extracted ion chromatograms of senkyunolide I and H in the dosed and control plasma in the negative ion mode. Figure S5. Difference between the chemical components or category and number of chemical components of Qi-Fu-Yin and the seven herbs. (A) Difference between the chemical components of Qi-Fu-Yin and the seven herbs. (B) Difference between the category and number of chemical components of Qi-Fu-Yin and the seven herbs. Table S1. Comparison between the current study and Li's study. [file 1995766.f1.zip › 1995766.f1/Supplementary Fig 3.jpg]

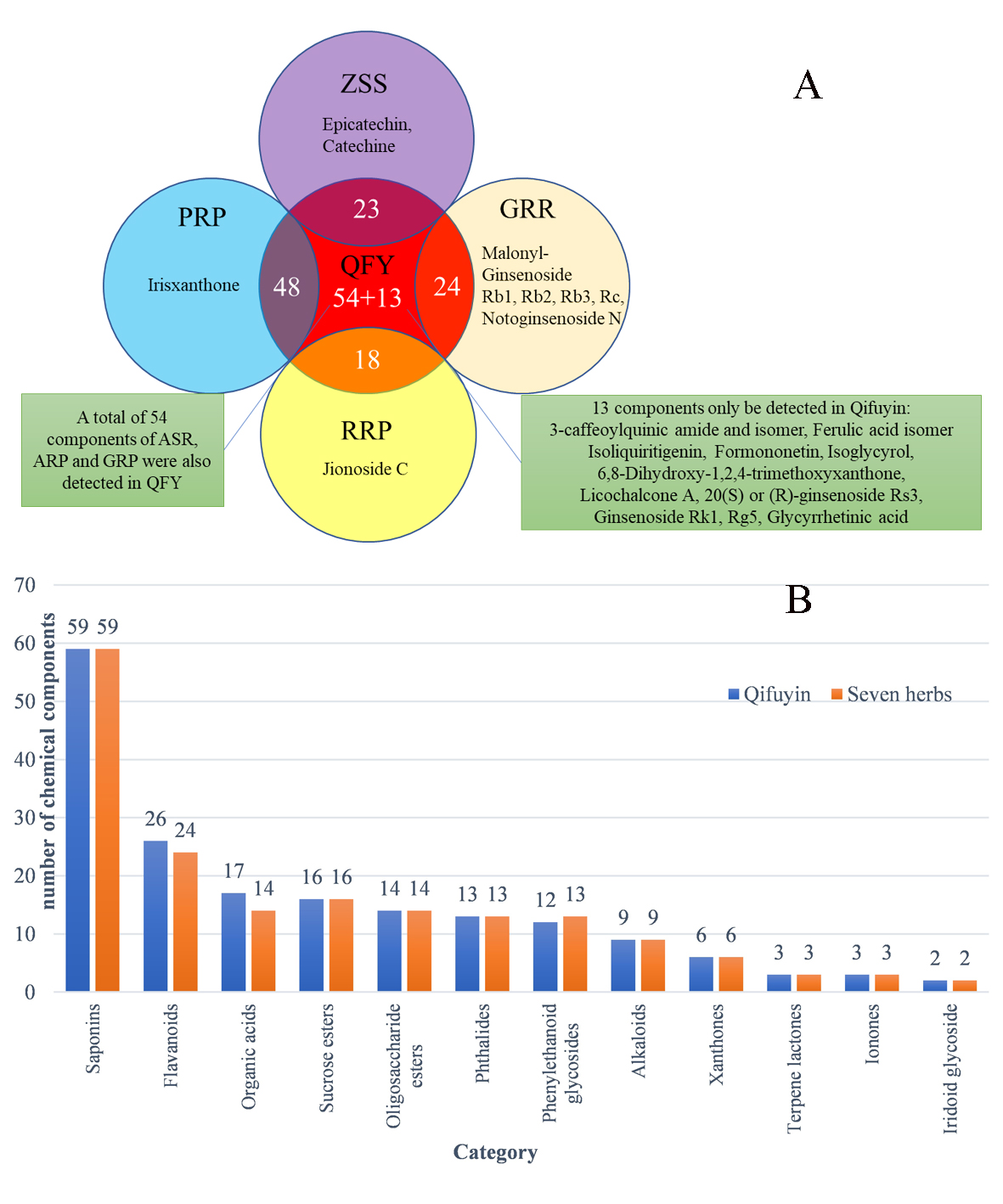

Supplement: Supplementary Materials — Figure S1. Base peak chromatograms of Qi-Fu-Yin and seven herbs in the positive (+) and negative (−) ion modes. QFY, Qi-Fu-Yin; GRR, Ginseng Radix et Rhizoma; RRP, Rehmanniae Radix Preparata; ASR, Angelicae Sinensis Radix; ARP, Atractylodis Macrocephala Rhizoma Preparata; GRP, Glycyrrhizae Radix et Rhizoma Preparata Cum Melle; ZSS, Ziziphi Spinosae Semen; PRP, Polygalae Radix Preparata. Figure S2. MS/MS spectra and the proposed fragmentation pathways of acteoside, schaftoside, and spinosyn. (A) MS/MS spectra and the proposed fragmentation pathways for acteoside. (B) MS/MS spectra and the proposed fragmentation pathways of schaftoside. (C) MS/MS spectra and the proposed fragmentation pathways for spinosin. Figure S3. MS/MS spectra and the proposed fragmentation pathways of tenuifoliside C. Figure S4. Extracted ion chromatograms of senkyunolide I and H in the dosed and control plasma in the negative ion mode. Figure S5. Difference between the chemical components or category and number of chemical components of Qi-Fu-Yin and the seven herbs. (A) Difference between the chemical components of Qi-Fu-Yin and the seven herbs. (B) Difference between the category and number of chemical components of Qi-Fu-Yin and the seven herbs. Table S1. Comparison between the current study and Li's study. [file 1995766.f1.zip › 1995766.f1/Supplementary Fig 4.jpg]

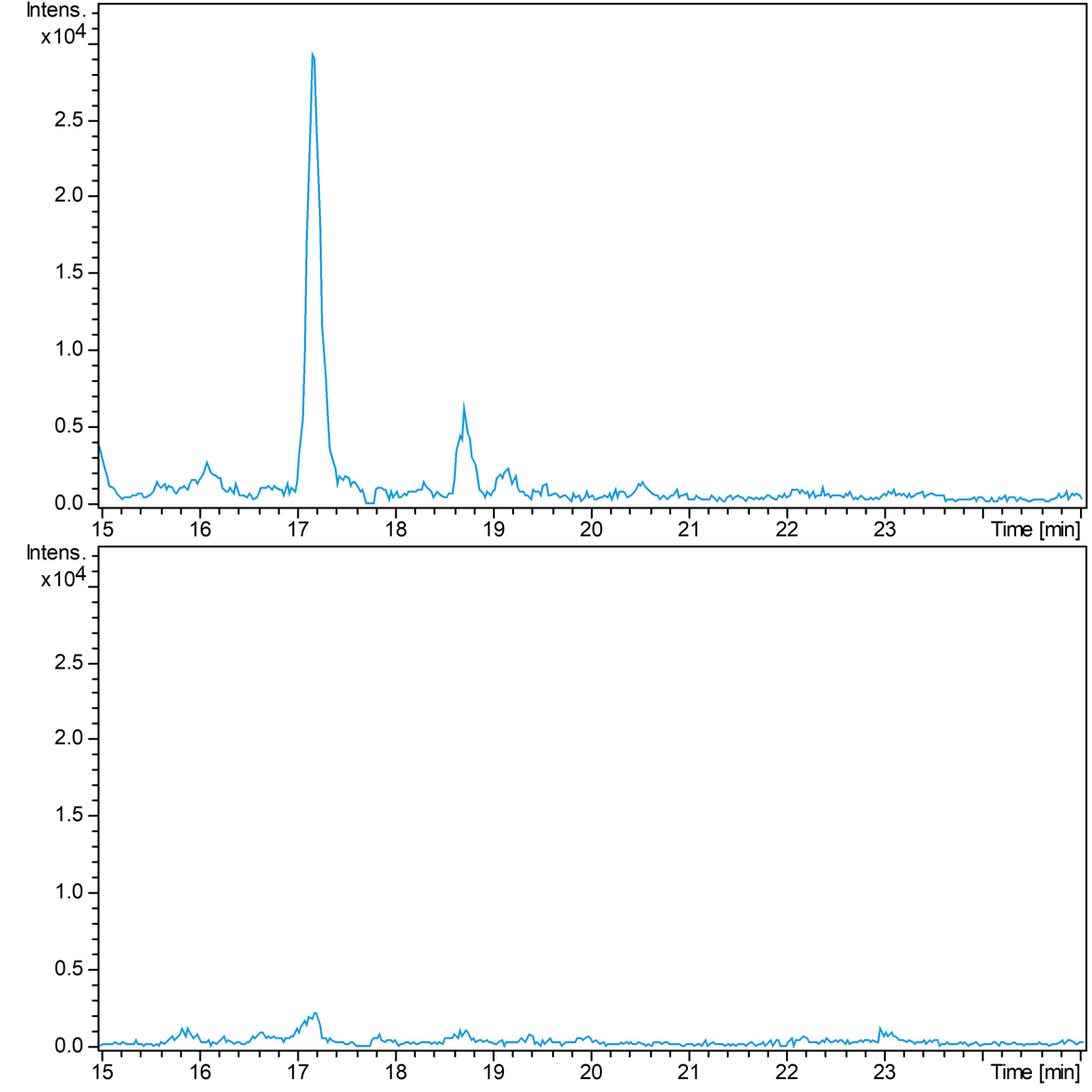

Supplement: Supplementary Materials — Figure S1. Base peak chromatograms of Qi-Fu-Yin and seven herbs in the positive (+) and negative (−) ion modes. QFY, Qi-Fu-Yin; GRR, Ginseng Radix et Rhizoma; RRP, Rehmanniae Radix Preparata; ASR, Angelicae Sinensis Radix; ARP, Atractylodis Macrocephala Rhizoma Preparata; GRP, Glycyrrhizae Radix et Rhizoma Preparata Cum Melle; ZSS, Ziziphi Spinosae Semen; PRP, Polygalae Radix Preparata. Figure S2. MS/MS spectra and the proposed fragmentation pathways of acteoside, schaftoside, and spinosyn. (A) MS/MS spectra and the proposed fragmentation pathways for acteoside. (B) MS/MS spectra and the proposed fragmentation pathways of schaftoside. (C) MS/MS spectra and the proposed fragmentation pathways for spinosin. Figure S3. MS/MS spectra and the proposed fragmentation pathways of tenuifoliside C. Figure S4. Extracted ion chromatograms of senkyunolide I and H in the dosed and control plasma in the negative ion mode. Figure S5. Difference between the chemical components or category and number of chemical components of Qi-Fu-Yin and the seven herbs. (A) Difference between the chemical components of Qi-Fu-Yin and the seven herbs. (B) Difference between the category and number of chemical components of Qi-Fu-Yin and the seven herbs. Table S1. Comparison between the current study and Li's study. [file 1995766.f1.zip › 1995766.f1/Supplementary Fig 5.jpg]
